# Supplementary material for: Behavior-Dependent Activity and Synaptic Organization of Septo-hippocampal GABAergic Neurons Selectively Targeting the Hippocampal CA3 Area
Source: Neuron. 2017 Dec 20;96(6):1342–1357.e5. doi: 10.1016/j.neuron.2017.10.033 (PMC5746169; doi:10.1016/j.neuron.2017.10.033)
Supplement: Document S1. Figures S1–S3 and Table S1 [file mmc1.pdf]

Neuron, Volume *96*

Supplemental Information

Behavior-Dependent Activity and Synaptic Organization of Septo-hippocampal GABAergic Neurons Selectively Targeting the Hippocampal CA3 Area

Abhilasha Joshi, Minas Salib, Tim James Viney, David Dupret, and Peter Somogyi

Supplemental Information

Cell ID	Target ID	Target Location	Domain	PV	SATB1	Endo-biotin	CCK	CB	SM	Figure
AJ42m	s47_d1	CA3	d	d+	u	u	u	u	u	
	s47_d2	CA3	d*	s, d +	n-	s+	s-	s-	s-	
	s47_d3	CA3	d*	s, d +	n+	s+	s-	s-	s-	
	s47_d4	CA3	d*	s, d +	n-	s+	s-	s-	s-	
	s47_d5	CA3	d	d+	u	u	u	u	u	
	s47_s1	CA3	s	s-	s-	s+	s+	s-	s-	5B
	s47_s2	CA3	s	s-	s-	s+	s+	s-	s-	5B
	s47_s3	CA3	s	s-	s-	s+	s+	s-	s-	
	s47_s4	CA3	s	s-	s-	s+	s+	s-	s-	
	s48_a1	CA3	s, d	s+	n-	s+	s-	u	u	S2 B
	s48_a4	CA3	s, d	s+	n-	s+	s-	u	u	5A
	s48_a8	CA3	s, d	s+	n-	s+	s-	u	u	5A
	s48_a9	CA3	s, d	s+	n-	s+	s-	u	u	5A
	s49_a1	CA3	s, d	s+	n-	s+	s-	u	u	S2 A
	s49_a2	CA3	s, d	s+	n-	s+	s-	u	u	
	s50_d1	CA3	d	d+	u	u	u	d-	u	
	s50_d2	CA3	d	d+	u	u	u	d-	u	
	s50_d3	CA3	d	d+	u	u	u	d-	u	
	s50_d4	CA3	d	d+	u	u	u	d-	u	
	s50_d5	CA3	d	d+	u	u	u	d-	u	
	s50_d7	CA3	d	d+	u	u	u	d-	u	
	s50_s1	CA3	s	s+	n-	s+	u	s-	u	S2 C
	s71_a10	MS	s	s+	u	s+	u	u	u	
	s71_a11	MS	s	s+	u	s+	u	u	u	
	s71_a9	MS	s, d	s, d +	n+	s+	u	u	u	3D
	s72_a1	MS	s, d	s, d +	n+	s+	u	u	u	
	s72_a2	MS	s, d	s, d +	n+	s+	u	u	u	
	s72_a3	MS	d	d+	u	u	u	u	u	
	s72_a4	MS	d	d+	u	u	u	u	u	
	s72_a5	MS	d	d-	u	u	u	u	u	
	s72_a6	MS	s	s+	n+	s+	u	u	u	
	s72_a7	MS	s	s+	n+	s+	u	u	u	
	s72_a8	MS	d	d+	u	u	u	u	u	
MS90g	s28_s1	CA3	s	s+	n-	s+	s-	u	u	
	s29_s1	CA3	s	s+	n-	s+	s-	u	u	
	s30_s1	CA3	s	s+	n-	s+	s-	u	u	
	s30_s2	CA3	s	s+	n-	s+	s-	u	u	
	s31_s2	CA3	sd	s+	n-	s+	s-	u	u	
	s32_s1	CA3	s	s+	n-	s+	s-	u	u	

Table S1. Subcortical and cortical synaptic target neurons of Teevra cells, AJ42m and MS90g. Molecular expression profiles of postsynaptic neurons, based on close apposition of septal terminals. '+' detectable positive immunoreactivity or signal; '-' undetectable immunoreactivity or signal in vicinity of immunopositive signals observed within subcellular domain: s, soma; n, nucleus; d, dendrite; d*, dendrite could be followed to the soma from which it originates. u, unknown (untested or unavailable).

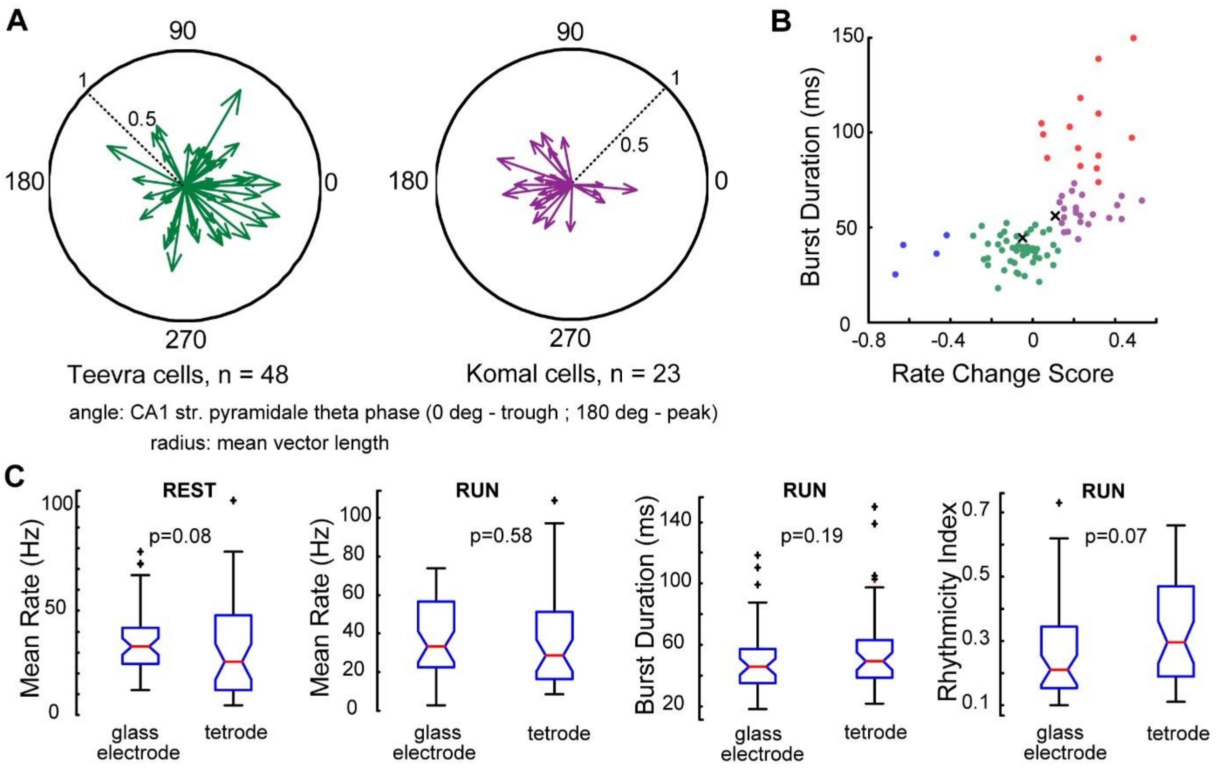

Figure S1: Physiological parameters of MS neurons recorded using glass-electrodes and silicon probes (related to Figure 1)

(A) Distribution of the mean theta firing phase of Teevra (green) and Komal (magenta) cells with mean vector length as radius. **(B)** Burst duration (ms) and rate change score of labelled Teevra neurons MS90g (x, left) and MS11b(x, right) in relation to other cells. These two neurons were not included in the clustering because the animals did not exhibit voluntary movement (see methods); the parameters were calculated during short bouts of theta oscillatory activity (Table 1). **(C)** Comparison of parameters calculated based on the spike train dynamics of neurons recorded using glass electrode or silicon probe recordings ($p > 0.05$, Kruskal-Wallis test). +, outliers.

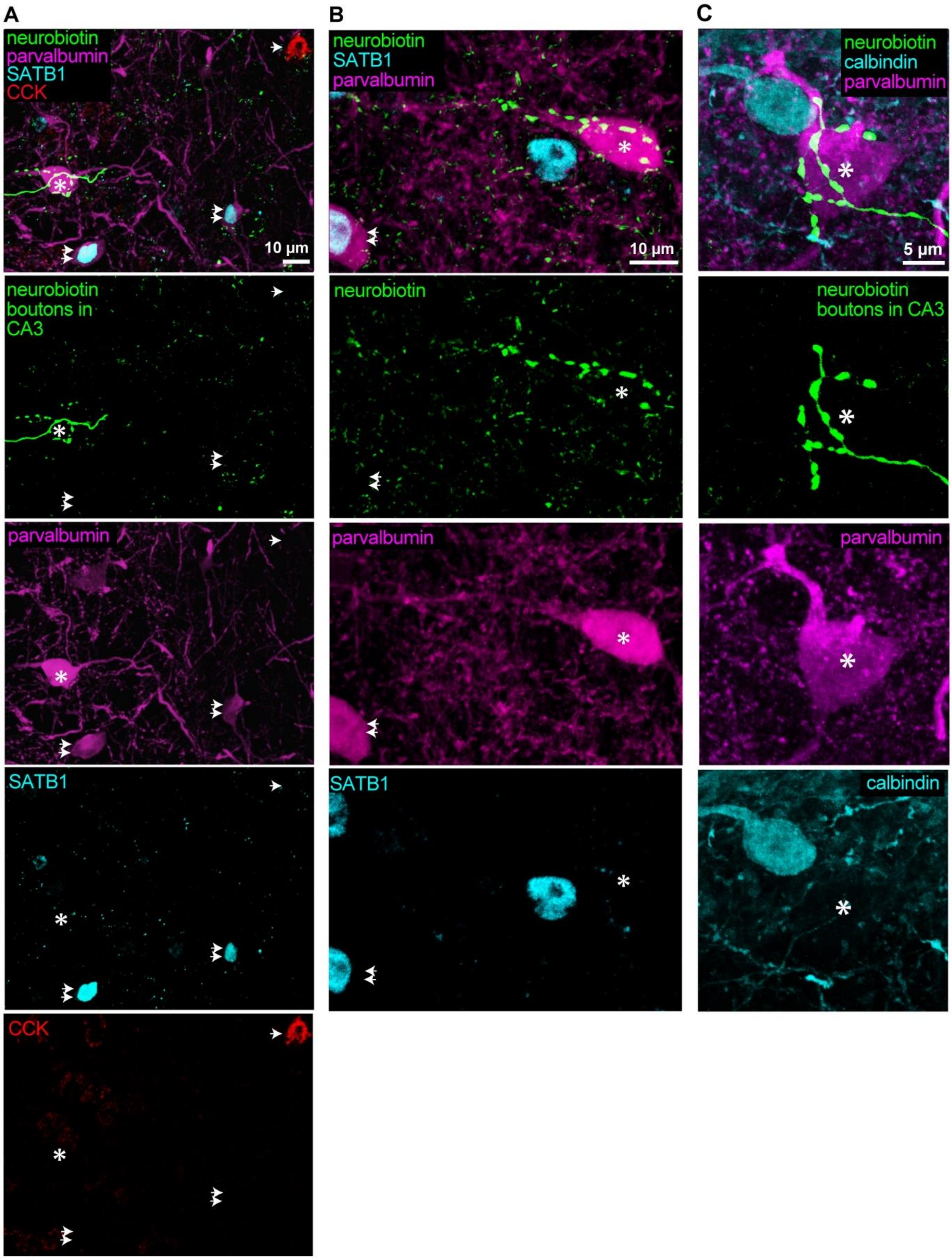

Figure S2: Postsynaptic PV+/SATB1-negative interneurons are consistently and preferentially innervated by Teevra cells. (related to Figure 5)

(A) Axonal terminals (green) of Teevra cell innervate PV+ (magenta, asterisks) and SATB1-negative (cyan) cells in a basket-like formation and also following their dendrites. Nearby PV+ and SATB1+ cells (double arrows) and a CCK+ cell (top, single arrow) were not innervated. **(B)** Axonal terminals (green) of Teevra cell innervate a PV^+ and SATB1-immunonegative cell in CA1 (labeling as in A). **(C)** Axonal terminals (green) of Teevra cell in CA3, innervate a PV+ (magenta, asterisk) and CB-negative (cyan) cell in a basket-like formation and also follow its dendrite, but do not innervate a neighboring CB^+ cell (cyan). The immunoreactivity of targeted interneurons is consistent with the molecular profile of axo-axonic cells. Imaging details (z-thickness in µm, z-projection type stated): (A) 9.74 µm, standard deviation projection (B) 11.7 µm, standard deviation projection (C) 14.70 µm, maximum intensity projection

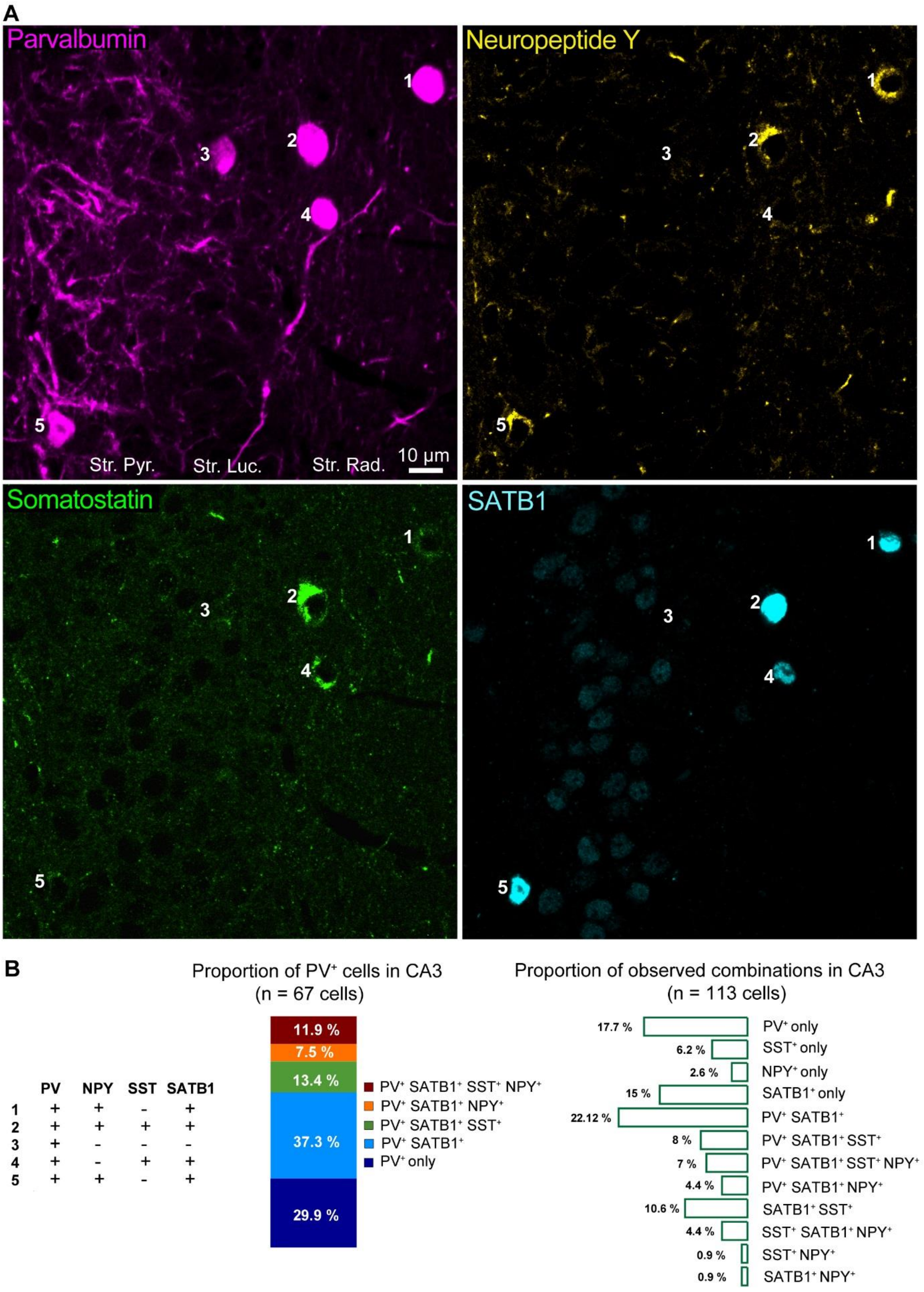

Figure S3: Molecular diversity of PV-expressing interneurons in mouse CA3 (related to Figure 5)

(A) A cluster of PV^+ interneurons (magenta, 1 to 5) exhibit a diverse combination of immunoreactivity for molecules expressed in strata pyramidale and lucidum of the mouse CA3. The SATB1-immunonegative nucleus of cell 3 is within the displayed optical slice and was also checked in other slices. **(B)** Left, immunoreactivity scores of the cells in 'A'. Middle, quantification of PV+ cells sampled with immunoreactivity for SATB1, NPY and SST (n = 3 mice, 5 areas). Note that PV-immunopositive and SATB1-immunonegative axo-axonic cells (Viney et al., 2013), comprise 30% of the PV+ population. All other PV+ cells also are SATB1+ in combination with other molecular markers such as SST and NPY. Right, proportions of interneurons labeled for combinations of the 4 molecules. Imaging details (z-thickness): 0.38 µm, single optical section.
