# Supplementary material for: Behavior-Dependent Activity and Synaptic Organization of Septo-hippocampal GABAergic Neurons Selectively Targeting the Hippocampal CA3 Area
Source: Neuron. 2017 Dec 20;96(6):1342–1357.e5. doi: 10.1016/j.neuron.2017.10.033 (PMC5746169; doi:10.1016/j.neuron.2017.10.033)
Supplement: Document S2. Article plus Supplemental Information [file mmc4.pdf]

Article

Neuron

Behavior-Dependent Activity and Synaptic Organization of Septo-hippocampal GABAergic Neurons Selectively Targeting the Hippocampal CA3 Area

Highlights

- Rhythmically firing medial septal neurons cluster into distinct subpopulations
- The most rhythmic GABAergic *Teevra* cells fire coincident with hippocampal excitation
- *Teevra* cells preferentially innervate CA3 axo-axonic and CCK GABAergic interneurons
- *Teevra* axons restricted to CA3 septo-temporal segments reflect degree of rhythmicity

Authors

Abhilasha Joshi, Minas Salib, Tim James Viney, David Dupret, Peter Somogyi

Correspondence

abhilasha.joshi@pharm.ox.ac.uk (A.J.), peter.somogyi@pharm.ox.ac.uk (P.S.)

In Brief

Using congruent neuronal features, Joshi et al. define the most rhythmic medial septal GABAergic cell type, the *Teevra* cells, which preferentially innervate axo-axonic GABAergic interneurons in the hippocampal CA3. Such selective termination coordinates hippocampal excitability and theta oscillations via disinhibition.

Joshi et al., 2017, Neuron *96*, 1342–1357
December 20, 2017 © 2017 The Authors. Published by Elsevier Inc.
https://doi.org/10.1016/j.neuron.2017.10.033

Behavior-Dependent Activity and Synaptic Organization of Septo-hippocampal GABAergic Neurons Selectively Targeting the Hippocampal CA3 Area

Abhilasha Joshi,[1,2,*] Minas Salib,[1] Tim James Viney,[1] David Dupret,[1,2] and Peter Somogyi[1,3,4,*]
[1]Department of Pharmacology, University of Oxford, Oxford OX1 3QT, UK
[2]MRC Brain Network Dynamics Unit, Department of Pharmacology, University of Oxford, Oxford OX1 3TH, UK
[3]Center for Brain Research, Medical University of Vienna, Vienna 1090, Austria
[4]Lead Contact
*Correspondence: abhilasha.joshi@pharm.ox.ac.uk (A.J.), peter.somogyi@pharm.ox.ac.uk (P.S.)
https://doi.org/10.1016/j.neuron.2017.10.033

SUMMARY

Rhythmic medial septal (MS) GABAergic input coordinates cortical theta oscillations. However, the rules of innervation of cortical cells and regions by diverse septal neurons are unknown. We report a specialized population of septal GABAergic neurons, the *Teevra cells*, selectively innervating the hippocampal CA3 area bypassing CA1, CA2, and the dentate gyrus. Parvalbumin-immunopositive Teevra cells show the highest rhythmicity among MS neurons and fire with short burst duration (median, 38 ms) preferentially at the trough of both CA1 theta and slow irregular oscillations, coincident with highest hippocampal excitability. Teevra cells synaptically target GABAergic axo-axonic and some CCK interneurons in restricted septo-temporal CA3 segments. The rhythmicity of their firing decreases from septal to temporal termination of individual axons. We hypothesize that Teevra neurons coordinate oscillatory activity across the septo-temporal axis, phasing the firing of specific CA3 interneurons, thereby contributing to the selection of pyramidal cell assemblies at the theta trough via disinhibition.

INTRODUCTION

Activity in the hippocampal CA1 area is spatially and temporally tuned during context-dependent behavior and the spiking of pyramidal cells and interneurons is organized within theta and gamma frequency oscillatory timescales. This temporal organization is supported by well-characterized glutamatergic projections from CA3 (Amaral and Witter, 1989; Middleton and McHugh, 2016) as well as from the entorhinal cortex (EC) (Brun et al., 2008; Witter et al., 1988). These inputs mediate both dendritic excitation and feedforward inhibition (Buzsáki 1984) of pyramidal cells. In addition to these cortical inputs, medial septal (MS) cholinergic (Gielow and Zaborszky, 2017), glutamatergic (Justus et al.,2017; Huh et al., 2010; Robinson et al., 2016; Fuhrmann et al., 2015), and GABAergic neurons innervating the hippocampus are part of a subcortical theta rhythm generating network involving the brainstem, thalamus, and hypothalamus (Vertes and Kocsis, 1997). Disruptions of MS input results in loss of theta power and impaired performance in spatial learning (Winson, 1978), disrupted learning in contextual fear conditioning (Calandreau et al., 2007), and a slowed rate of acquisition of delayed eyeblink conditioning (Berry and Thompson, 1979). A striking yet underappreciated feature of GABAergic septal afferents to the hippocampus is the extensive targeting of interneurons in CA3 and the hilus and granule layer of the dentate gyrus (DG) compared to CA1 (Freund and Antal, 1988). The key role of CA3 pyramidal cells in the hippocampal circuit is underlined by their bilateral projections, topographically organized through highly interconnected cell assemblies and providing the numerically largest innervation to CA1 (Witter, 2007). Interestingly, CA3 inactivation does not hamper rate coding in CA1; however, it is required for the emergence of theta sequences (Foster and Wilson, 2007; Middleton and McHugh, 2016). Furthermore, the CA3 area and the DG are likely to be involved in distinct aspects of spatial coding (Neunuebel and Knierim, 2014) raising the hypothesis that septal GABAergic inputs to the hippocampal subsystems might have distinct connectional and temporal organization. However, the organization of MS inputs to hippocampal or cortical areas at single-cell resolution are largely unknown.

In CA3-CA1, pyramidal cells are active at the trough relative to dorsal hippocampal CA1 theta oscillations (Csicsvari et al., 1999; Lasztóczi et al., 2011). This activity is coordinated by diverse local interneurons of the hippocampus, which provide temporally coordinated rhythmic inhibition to distinct pyramidal subcellular compartments (Somogyi et al., 2013). Some of these GABAergic interneurons, e.g., axo-axonic cells (Viney et al., 2013), do not fire when the pyramidal cells are most excitable, whereas others (e.g., bistratified and O-LM cells, Katona et al., 2014) fire maximally together with the overall population of pyramidal cells. The way these differences among GABAergic interneurons are brought about in the network is beginning to emerge from analysis of their long range synaptic inputs (Leão et al., 2012; Fuhrmann et al., 2015; Kaifosh et al., 2013). A key missing link is the theta

 © 2017 The Authors. Published by Elsevier Inc.
This is an open access article under the CC BY license (http://creativecommons.org/licenses/by/4.0/).

firing-phase preferences of subcortical inputs to defined types of hippocampal interneuron.

Do septo-cortical long-range projection neurons follow target-region-specific axonal distributions and cell-type-specific theta-phase firing preferences? In order to define the contribution of septal inputs at single-cell resolution, we set out to determine whether rhythmic septal neurons with similar activity patterns project to the same or distinct hippocampal areas. We used a combination of extracellular multiunit recordings, targeted single-neuron recording, and juxtacellular labeling (Pinault, 1996) in behaving head-fixed mice to reveal the rules of septo-hippocampal connectivity. Here, we report the activity patterns of a distinct group of rhythmic MS GABAergic neurons, named "Teevra cells," which selectively target interneurons in spatially restricted domains of the CA3 region of the hippocampus but do not innervate DG or CA2 and only minimally CA1. We have determined their molecular profiles, synaptic partners, and organizational principles along the hippocampal septo-temporal axis.

RESULTS

Subpopulations of MS Rhythmic Neurons Based on Spike Train Dynamics: *Teevra* and *Komal* Neurons

Using multichannel extracellular probes, we recorded neuronal activity in the septum of head-fixed mice (n = 7) during running (RUN) and pauses (REST) while they navigated on a virtual linear maze. The location of the probe and recording sites were established histologically in fixed brain sections *post hoc*, and further analysis was restricted to the cases where several recording sites were confirmed to be in the medial septum (MS) (n = 4 mice, Figure 1A). The action potential firing frequency of recorded neurons in the MS during running varied widely (median: 23.98 Hz; interquartile range [IQR]: 13.4–38.5 Hz, n = 81 neurons) and was higher than adjacent lateral septal (LS) neurons (median: 2.55 Hz, IQR: 1–7.1 Hz, n = 18 neurons; Kruskal-Wallis test, $p < 10^{-8}$). All MS neurons recorded in this configuration were phase coupled to ongoing theta oscillations recorded in dorsal hippocampal CA1, whereas this was the case only for 27% of LS neurons (Rayleigh test, $p < 0.05$). Thus, MS neurons differed from adjacent LS neurons both by their firing rate during locomotion and phase coupling to local field potential (LFP) theta oscillations in CA1.

Rhythmic burst firing is considered to be a characteristic feature of MS GABAergic neurons (Borhegyi et al., 2004; Dragoi et al., 1999; King et al., 1998; Simon et al., 2006). We observed a striking diversity in the burst duration and extent of rhythmicity of action potential firing among simultaneously recorded MS neurons (Figure 1A). To capture this, we estimated the burst duration (see STAR Methods) and calculated a rhythmicity index (RI; see STAR Methods), which bounded from 0 to 1 in order of increasing rhythmicity. During RUN periods, simultaneously recorded MS neurons (range: 5 to 19) exhibited varying burst duration (median: 55 ms, IQR: 40.6–87.9 ms, n = 81 neurons) and extent of rhythmicity (median: 0.13, IQR: 0.04–0.31, n = 81 neurons). Additionally, the preferential mean firing phase of the individual cells, collectively, covered the entire theta cycle as referenced to ongoing LFP theta oscillations in the pyramidal layer of CA1. We further found that simultaneously recorded individual MS neurons could increase, decrease, or not change their mean firing rate between REST and RUN periods, and this was consistent for a given cell for different periods of the recording session. In order to capture this behavioral state dependence, we computed a rate change score from REST to RUN, which bounded from −1 to 1 (see STAR Methods).

Similar activity dynamics could be identified and measured from extracellular glass electrode recordings of single MS neurons in behaving head-fixed mice (n = 65 neurons, N = 24 mice). Further analysis was restricted to MS neurons with a rhythmicity index >0.1 from both recording configurations (n = 43 tetrode, n = 46 glass electrode). We have calculated the rate change score and burst duration for all neurons (n = 89) and fed them to an unsupervised hierarchical clustering algorithm to explore the characteristics of major clusters (Figure 1B).

The most populous cluster of MS neurons (group 2; n = 48, mean silhouette value: 0.74) exhibited negligible change in their firing rate from REST to RUN (median rate change score: −0.05, IQR: −0.14–0.005), a high firing rate during RUN (median: 30.5 Hz, IQR: 22.8–51.4 Hz), and a short burst duration (median: 38 ms, IQR: 33.5–42.2 ms, Figure 1C). When we converted the burst of action potential waveforms to sound, these neurons had a "sharp" vocalization; therefore, we have named these *Teevra* cells (e.g., neuron aj27b_10 in Figure 1C; Movie S1 and explanation). In contrast, MS neurons in the second largest cluster (group 3; n = 23, mean silhouette value: 0.74) increased their firing rate from REST to RUN (median rate change score: 0.21, IQR: 0.16–0.29), had a high firing rate during RUN (median: 41.5 Hz, IQR: 30.6–62.9 Hz), and had a long burst duration (median: 57 ms, IQR: 53.4–64 ms, Figure 1C); we have named these *Komal* cells based on the "soft" or "flat" sound of the burst (e.g., neuron aj27b_9 in Figure 1C; see also Movie S1). Teevra and Komal neurons differed in their burst duration during RUN ($p = 8.7 \times 10^{-11}$, Kruskal-Wallis test) and in the firing rate change score ($p = 1.2 \times 10^{-11}$, Kruskal-Wallis test), but their mean firing rate during running periods was not different (p = 0.12, Kruskal-Wallis test). The activity of the two groups of neurons recorded by tetrodes also differed in their correlation with running speed, which was measured by a linear correlation coefficient "r" (Teevra cells, median "r": −0.02, IQR: −0.14–0.11, n = 21; Komal cells, median r: 0.37, IQR: 0.21–0.53, n = 12; $p = 1.5 \times 10^{-5}$, Kruskal-Wallis test), individual examples are shown in Figure 1C. In addition to the two largest groups, group 1 neurons (n = 4) decreased firing from REST to RUN and had a low mean firing rate during RUN (median: 7 Hz, IQR: 4–13 Hz), and group 4 neurons (n = 14) increased their firing rate from REST to RUN (median rate change score: 0.27, IQR: 0.18–0.32) and had a low firing rate during RUN (median: 14.5 Hz, IQR: 13.2–37.1 Hz).

The mean firing-phase preference of septal neurons with respect to ongoing theta oscillations recorded in dorsal CA1 provides information about possible temporal specializations in their activity and influence. We tested whether Teevra and Komal neurons were different in the mean firing-phase preference relative to CA1 theta, a parameter not used in the clustering. The pooled firing-phase preferences of Teevra and Komal neurons were

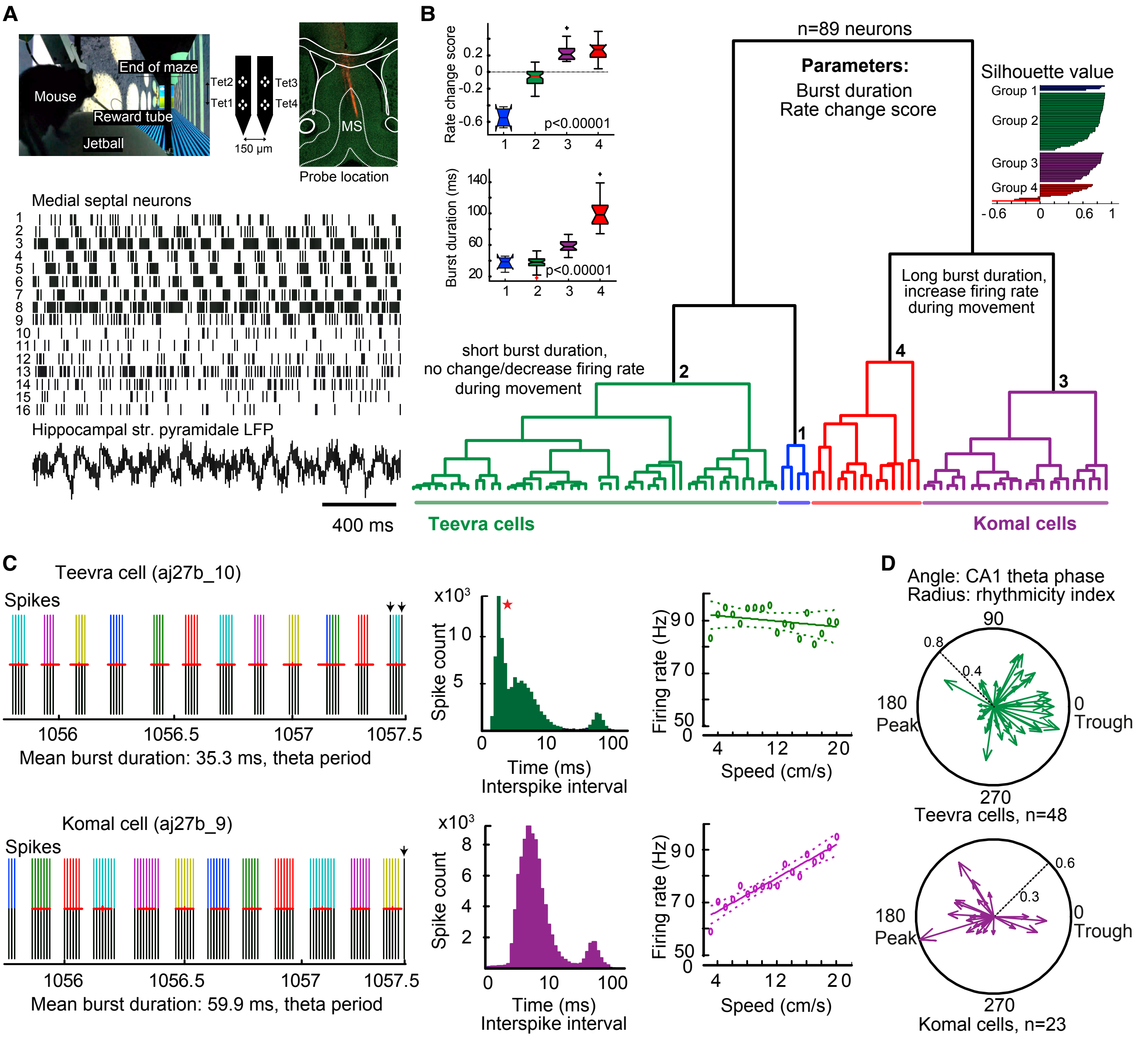

Figure 1. Subpopulations of MS Rhythmic Neurons Based on Spike Train Dynamics

(A) Simultaneously recorded septal neurons display diverse firing patterns (bottom, ticks) in head-fixed mice running on a Styrofoam ball (top, left). MS neurons were sampled with a silicon probe (top, middle). Note, DiI painted silicon probe depicts recording location in the MS (top, right).

(B) Hierarchical clustering of strongly rhythmic MS neurons (rhythmicity index > 0.1, n = 89) into four groups based on rate change score and burst duration as parameters. Left, comparison of rate change score and burst duration for the four groups (median values, Kruskal-Wallis test). Right, silhouette values show high intra-cluster similarity. Cells in each cluster are ordered by decreasing silhouette value (range, −1 to 1). Large positive values indicate group cohesion for each point (cell) toward points in its own cluster versus points in other clusters (see STAR Methods for calculation).

(C) Representative simultaneously recorded Teevra and Komal cells show differences in their burst duration. Left, during theta oscillations (s), detected bursts are shorter for Teevra cell (red horizontal lines, bursts detected; colors identify spikes within a burst; arrows, spikes not detected by the algorithm). Middle, inter-spike interval histogram of Teevra cell displays an additional peak at <5 ms (asterisk) compared to Komal cell. Right, the firing rate of a Teevra cell is not modulated by running speed (green, weighted fitting function $f = 92.6\ \text{Hz} - 0.26 \times s$; where s is the speed), whereas that of a Komal cell increases with running speed (purple, $p < 0.005$, weighted fitting function $f = 60.5\ \text{Hz} + 1.6 \times s$; where s is the speed, dashed lines, 95% confidence interval).

(D) Preferential theta phase of firing of Teevra and Komal cells with rhythmicity index as the radius (RUN periods). Most Komal cells (purple) fire preferentially at the peak of CA1 pyramidal cell layer theta oscillations, whereas most Teevra cells (green) fire phase coupled to the trough with increasing rhythmicity index. See also Movie S1 and explanation and Figure S2.

Table 1. Physiological Parameters and Immunohistochemical Analyses of Identified Septo-CA3 Projecting Teevra Neurons

Teevra	Rate (Hz)		Rate Change	Mean Burst	Phase Preference	Rhythmicity			Immunoreactivity			
Cell ID	Rest	Run	Score	Duration (ms)	(deg), dorsal CA1	RI	OF (Hz)	Target Area	PV	SATB1	mGluR1a	VGAT
AJ42m[R]	72.4	61.6	−0.1	37.9	341.6	0.73	7.5	CA3/(CA1)[R]	a+	n+	s−	t+
AJ37z	46.6	40.1	−0.1	25.8	7.0	0.61	7.2	CA3[L]	u	u	u	u
AJ45h[L]	28.5	33.4	0.1	49.1	5.7	0.48	6.8	CA3/(CA1)[L]	s+	n+	s−	t+
AJ43n	67.1	63.1	0.0	46.9	320.5	0.43	7.4	u	d+	n+	s−	t+
MS31h	20.5	20.6	0.0	39.1	115.0	0.30	6.2	CA3[L]	u	u	u	u
AJ40y[R]	30.9	24.6	−0.1	32.4	121.3	0.28	6.9	CA3[R]	d+	n+	s−	u
AJ48j[L]	38.9	33.2	−0.1	26.3	83.6	0.26	6.8	CA3[L]	s+	n+	d−	t+
AJ52g[R]	27.0	27.6	0.0	51.6	357.0	0.14	7.4	CA3[R]	s+	n+	s−	u
AJ43l[R]	28.6	23.4	−0.1	39.4	52.3	0.10	7.7	u	s, a−	n+	s+	u
MS11b[a]	31.8	39.7	0.1	56.0	320.3	0.01	6.1	CA3[L]	u	u	d−	u
MS71c[L]	47.6	49.4	0.0	42.3	34.1	0.14	7.1	CA3[L]	s+	n+	s−	u
MS73c[L]	52.5	34.6	−0.2	36.0	75.0	0.23	6.3	CA3[L]	s+	n+	s−	u
MS90g[a]	36.3	32.7	0.05	44.5	138.6	0.29	7.9	CA3/(CA1)[R]	a+	n+	s−	u

Positive (+) or undetectable (−) immunoreactivity within subcellular domain: s, soma; n, nucleus; d, dendrite; a, axon; t, axon terminals; u (unavailable or inconclusive). Not shown: AJ42m, AJ43l, AJ45h, AJ48j, AJ52 g are immunonegative for NK1 receptor (s, d) and AJ42m, AJ43l, AJ48j, AJ45h are immunonegative for vGlut2 (t). (CA1), small extent. Superscripts denote location of soma and axonal terminal in the right [(R)] or left [(L)] hemisphere. RI, rhythmicity index; OF, oscillatory frequency, RUN periods.

[a]Animal was not trained. These cells were not included in clustering due to absence of voluntary movement.

significantly different (Figures 1D and S1; $p < 0.002$, Watson's U^2 test, difference of circular means = 160°), with most Teevra neurons firing preferentially around the trough while most Komal neurons preferring the peak of dorsal CA1 stratum pyramidale theta LFP. Note that within both groups there are individual neurons with diverse firing-phase preferences. For Teevra cells, the trough phase preference correlated with a higher rhythmicity index (angular-linear correlation coefficient: 0.49, $p = 0.003$, $n = 48$, Figure 1D).

Rhythmic Activity of Teevra Cells Is Coincident with Heightened CA1 Excitation

Having identified distinct groups of MS neurons based on activity dynamics, we selected the largest group, the Teevra cells, which had the highest rhythmicity index (median: 0.3, IQR: 0.18–0.55, n = 48), for testing the hypothesis that these neurons represent a distinct population in the septo-cortical circuit. The rhythmicity indices of the other groups were group 1 (median: 0.19, IQR: 0.1–0.3, n = 4), group 3 (median: 0.19, IQR: 0.15–0.32, n = 23); group 4 (median: 0.19, IQR: 0.12–0.29, n = 14) ($p = 0.039$, 4 groups, Kruskal-Wallis test). The identification of Teevra cells was achieved by single-unit extracellular recording for cell selection based on firing patterns, and subsequent juxtacellular labeling (n = 13; Table 1) to aid their visualization and anatomical analysis (Figure 2A).

Hippocampal circuit activity is known to be influenced by the behavioral state of the animal, a feature thought to reflect particular stage of information processing. To assess the contribution of Teevra cells to the hippocampal circuit, first we evaluated the behavioral state dependent change in rhythmicity index from REST to RUN periods. We found that Teevra cells maintained rhythmic bursts discharge during both REST and RUN periods (Figures 2B and 2C), but their rhythmicity increased during RUN (median rhythmicity index rest: 0.07, IQR: 0.04–0.1; median rhythmicity index run: 0.3, IQR: 0.18–0.55, Wilcoxon paired-sample test, $p = 1.6 \times 10^{-09}$). Consistent with the increase in theta frequency during running (Sławińska and Kasicki, 1998), the oscillatory frequency (OF) of Teevra cells also increased during RUN (median oscillatory frequency rest: 6.4 Hz, IQR: 6.04–6.5 Hz; median oscillatory frequency run: 7 Hz, IQR: 7–7.34 Hz, Wilcoxon paired-sample test, $p = 4.9 \times 10^{-07}$). Examples of juxtacellularly labeled Teevra neurons AJ42m and AJ45h (Figures 2B and 2C) show such an increase in rhythmicity index and in the oscillatory frequency of firing. The bursts of Teevra cells often started on the descending phase of CA1 theta in line with the reported increase in CA3 pyramidal cell firing (Mizuseki et al., 2009).

Next, we explored the activity patterns of Teevra cells during bouts of REST periods when the hippocampal CA1 field potential was dominated by large amplitude irregular activity (LIA). Interestingly, firing of Teevra cells coupled to the falling transition of the LIA following the variable duration of the slow cycles (Figure 2E) and mirroring the theta trough coupling during RUN. Thus, during both REST and RUN, Teevra cells become active at times of LFP troughs, coincident with heightened excitation in CA1 (Mizuseki et al., 2009). Their bursting follows the frequency at which LFP troughs occur both during regular theta oscillations and the irregular slower waves at 2–6 Hz, which are accompanied by high-frequency bursts of Teevra cells at the negative phase of the wave.

In rats, MS GABAergic cells could be active or inhibited during sharp wave ripple (SWR) oscillations (Borhegyi et al., 2004; Dragoi et al., 1999; Viney et al., 2013). Under our behavioral paradigm of head-fixed mice, sharp wave ripples (130–240 Hz) were infrequent but could be observed for some Teevra cells (n = 4), which did not change their firing significantly during ripple events (Figure 2F) ($p > 0.05$, two-sample KS test, Katona et al., 2014).

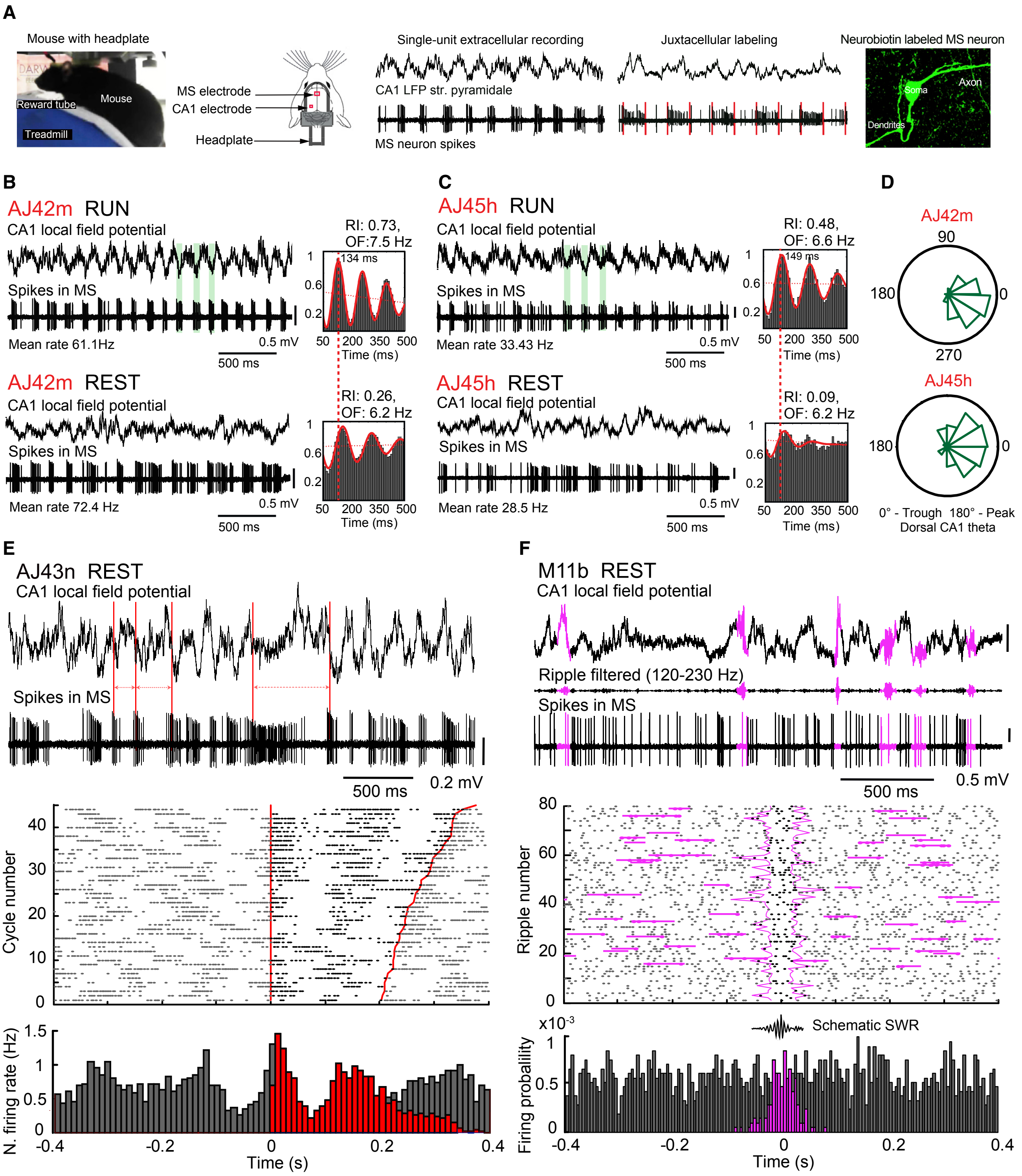

Figure 2. Teevra Neurons Fire Rhythmically and Coincident with Increased Hippocampal CA1 Excitation

(A) Single MS neurons and CA1 LFP were recorded extracellularly using glass electrodes in mice alternating between REST and RUN periods on a spherical treadmill. Teevra neurons preselected based on the sharp burst duration were juxtacellularly labeled with neurobiotin (green).

(B and C) Rhythmic burst firing of Teevra cells AJ42m (B) and AJ45h (C) during RUN (top) and REST (bottom). Local field potential (LFP) in CA1 stratum pyramidale. Some of the theta trough-centered bursts of MS neuron action potentials relative to CA1 theta oscillations are highlighted (light green). The autocorrelograms show a slight increase in oscillatory firing frequency (OF) and large increase in the rhythmicity index (RI) during RUN (top) compared to REST (bottom).

(legend continued on next page)

Teevra Cells Are GABAergic and Immunopositive for Parvalbumin and SATB1 but Not for mGluR1a in the Somatic Membrane

Teevra cells comprised a distinct subpopulation of MS neurons based on physiological parameters. Next, we have tested whether they represent a distinct cell type according to molecular markers and transmitter phenotype. Labeled Teevra cells were immunopositive for the calcium binding protein parvalbumin (PV, Figure 3A) (n = 9/10 tested), the transcription factor SATB1 (Figure 3B, n = 10/10 tested), but lacked detectable immunoreactivity for mGluR1a in the somatic plasma membrane (n = 10/11 tested). A weak cytoplasmic signal may represent a pool of receptor in the endoplasmic reticulum. Approximately half of the PV^+ neurons were immunopositive for SATB1 in the entire MS complex (unpublished data) and showed all four possible combinations of immunoreactivity for PV and mGluR1a, including double-immunonegative neurons. This indicates a differentiation among various GABAergic MS neurons to be defined in future studies with respect to their projections. Labeled Teevra cells were also tested for molecular phenotype of their boutons; all tested cells (n = 4/4; Table 1) were immunopositive for VGAT but not VGlut2 (Figure 3C) confirming that they were GABAergic and not glutamatergic neurons.

Teevra cells emitted axonal collaterals and boutons in the MS (n = 5/5 tested). These collaterals targeted mainly PV^+ soma or dendrites (n = 10/11 tested targets, AJ42m axon, Figure 3D; Table S1). All of the tested neurons innervated on their soma in the MS were PV^+ and $SATB1^+$ (n = 5/5). Varicosities of the axon of Teevra cell AJ42m were tested for the presence of synapses using labeling for the postsynaptic junction scaffolding protein gephyrin (Lardi-Studler et al., 2007), and the majority of boutons formed GABAergic synapses (n = 42/47 tested varicosities, Figure 3D). The identity of the PV^+ MS neurons innervated by Teevra cells remains to be determined. They may be other Teevra cells synchronized by local interconnections (Leão et al., 2015) or GABAergic MS neurons projecting to other cortical areas or different types of interneuron as suggested by Borhegyi et al. (2004).

GABAergic Teevra Cells Preferentially Innervate the CA3 and Target PV^+ Axo-Axonic Cells as well as CCK^+ Interneurons

MS GABAergic neurons innervate all hippocampal regions and many extra-hippocampal cortices (Freund and Antal, 1988; Unal et al., 2015), though it is not known whether single neurons innervate one or multiple cortical areas. We are unaware of the target area visualization of single GABAergic septal neurons with known activity patterns in the literature. Accordingly, to explain the basis of the influence of Teevra cells on cortical activity, we tested the distribution of their axonal terminals. All labeled Teevra cells projected either the left or the right hippocampus. Among the labeled Teevra cells whose axon could be followed to branches in the gray matter (n = 11/13), all innervated the CA3 region of the hippocampus preferentially, and no branches or varicosities were observed in the DG or CA2 (Figure 3E and 4; Table 1). The axons of Teevra cells traveled to the hippocampus either via the dorsal fornix (n = 2) or the fimbria (n = 11). In CA3, Teevra cells innervated interneurons (Figure 4). Of a total of 472 sampled boutons from 12 coronal hippocampal sections (n = 3 cells, section thickness 70–80 μm), 91.5% of boutons were in CA3 (n = 432), and only 8.5% boutons were observed in CA1 (n = 40). The main axon terminated in the hippocampus and no branches were observed in the retrosplenial cortex, the subiculum, the pre- and para-subiculum, or the entorhinal cortex. This preferential termination in CA3 was accompanied by a septo-temporal specialization of axonal branching with collaterals innervating only a restricted septo-temporal domain in CA3. For single Teevra cells, the majority of axonal branches and boutons were observed only through 5–8 coronal sections (section thickness: 70–80 μm). Although we cannot exclude the possibility of incomplete labeling, most terminal axon collaterals ended in boutons indicating a restricted area of termination. The most sensitive axon visualization method of horseradish peroxidase (HRP) reaction following freeze-thaw permeabilization and diaminobenzidine (DAB) reaction end-product intensification with osmium (see STAR Methods) was applied to the full course of labeled axons to detect potential collateral branches. It is unlikely that we missed substantial projections to CA1. The intrahippocampal spatial positions of the collaterals of the least rhythmic labeled Teevra cell in temporal CA3 (MS11b) did not overlap at all with those of the most rhythmic neuron (AJ42m) in septal CA3, showing the change in rhythmicity together with spatial progression along the septo-temporal extent of the hippocampal formation.

To test the synaptic targets of Teevra cells in CA3, we analyzed two labeled Teevra cells (AJ42m and MS90g). The targets of the few boutons encountered in CA1 were not tested. The axon of AJ42m was the most strongly labeled as its collateral branches could be followed to terminal boutons throughout the axonal arbor in CA3, both by fluorescence microscopy and following HRP reaction. We determined the molecular characteristics of 22 cellular target profiles (Table S1). In CA3, 18 out of 22 tested targets were PV^+, 11 were dendrites, and 7 somata. The PV^+ somatic profiles were all SATB1-immunonegative neurons

(D) Preferential coupling of spikes to CA1 theta troughs during RUN.

(E) Rhythmic burst firing of a Teevra neuron (AJ43n) during REST, a period dominated by large amplitude irregular activity (LIA) in CA1 stratum pyramidale (top). Consecutive zero crossings at falling transition of the LFP (red lines) are marked. Spike raster plot (middle) and normalized spike firing histogram (bottom) show correlation of spikes (gray dots) with the timing of slow LFP oscillation cycles. Consecutive LFP zero crossings (>200 ms apart) are ordered according to their duration and marked by red lines; spikes identified within two consecutive cycles are colored black; time 0 is the zero crossing of LFP falling transitions. Note additional burst at ~6 Hz between time points marked by red lines.

(F) During ripple oscillations (pink, top), a Teevra neuron (MS11b) does not change its firing probability. Spike raster plot (middle) and firing probability (bottom) show sustained activity during ripple epochs (pink bars in the histogram) compared to ±0.4 s (gray) surrounding the peak of sharp wave ripple events. Raster plots were aligned to the peak sharp wave ripple power, and pink lines delineate the beginnings and end of sharp wave ripples; spikes within the sharp wave ripple period are colored black. Time 0 is the peak power of each ripple oscillatory event.

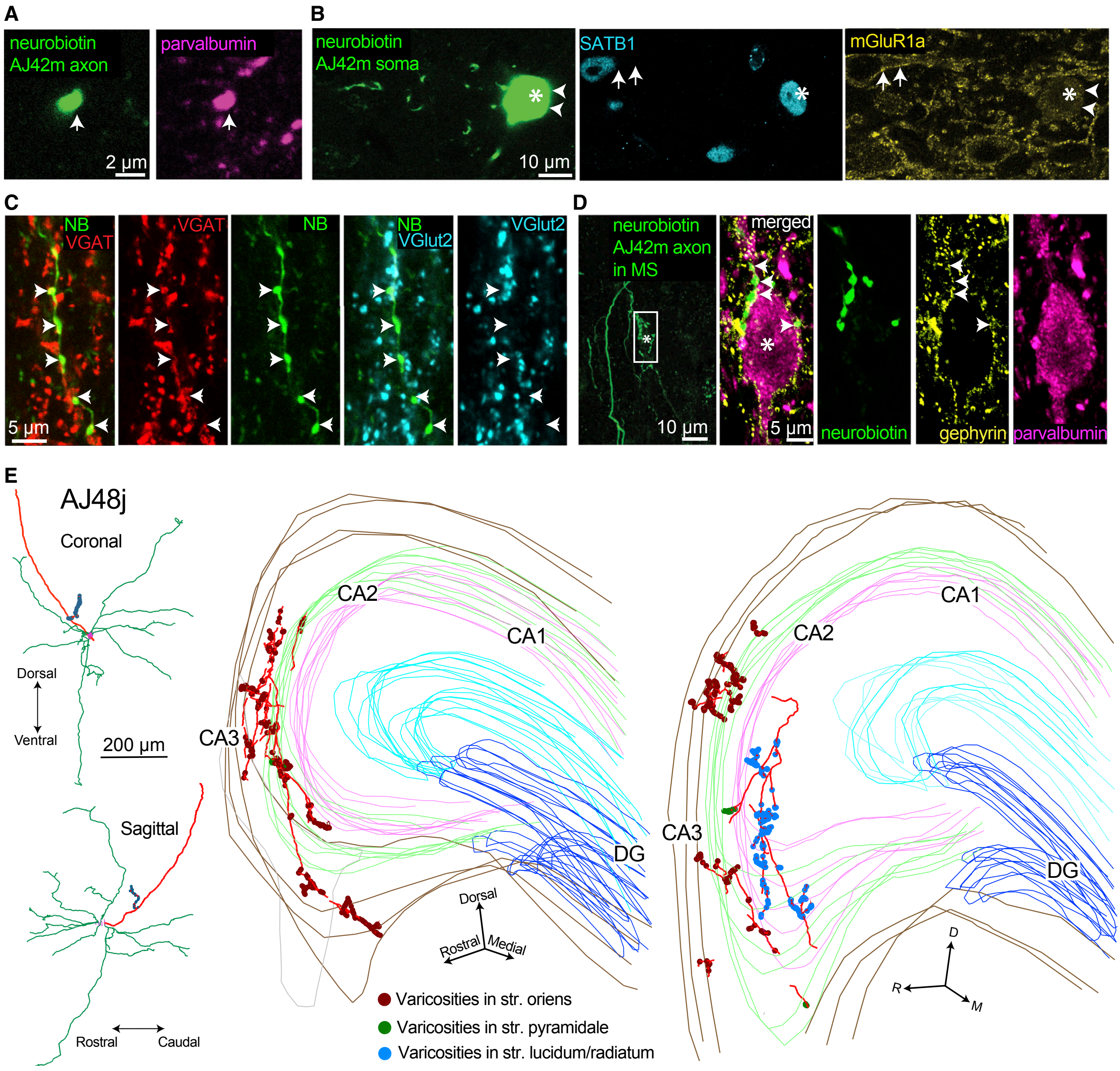

Figure 3. Teevra Neurons Are GABAergic, Innervate PV+ Neurons in the Septum, and Target the Hippocampal CA3 Region

(A and B) Neurobiotin-labeled Teevra neuron; AJ42m was PV+ (A) (magenta, main axon), SATB1+ (B) (cyan, asterisk, nucleus), and immunonegative for mGluR1a in the plasma membrane (arrowheads) showing only a weak cytoplasmic signal. Note, mGluR1a+ somatic membrane labeling (yellow, arrows) of a neighboring SATB1+ cell is shown.

(C) Axon terminals of AJ42m (green) were VGAT+ (red, arrows) and VGlut2− (cyan, arrows).

(D) Main axon and local terminals of AJ42m in the MS (green, box) innervating a PV+ soma (magenta, asterisk) in a basket-like formation. Note, gephyrin puncta (yellow, arrows) outlining the somato-dendritic membrane.

(E) Left, reconstruction of Teevra cell AJ48j in coronal and sagittal views showing a complete ovoid dendritic field (green), soma and main axon (red), and a local axonal branch with boutons (blue). Right, the axonal varicosities (100× objective, color coded by layer) of AJ48j in two series of consecutive 80-μm-thick sections (left, 4 sections; right, 3 sections), showing preferential termination in part of CA3. Coronal sections are rotated to highlight laminar selectivity (D, dorsal; M, medial, R. rostral).

Imaging details (z-thickness in micrometers, single optical slices unless z-projection type stated): (A) 0.60 μm, (B) 0.32 μm, (C) 0.70 μm, maximum intensity projection (D, left) 33.60 μm, maximum intensity projection (D, right) 0.37 μm.

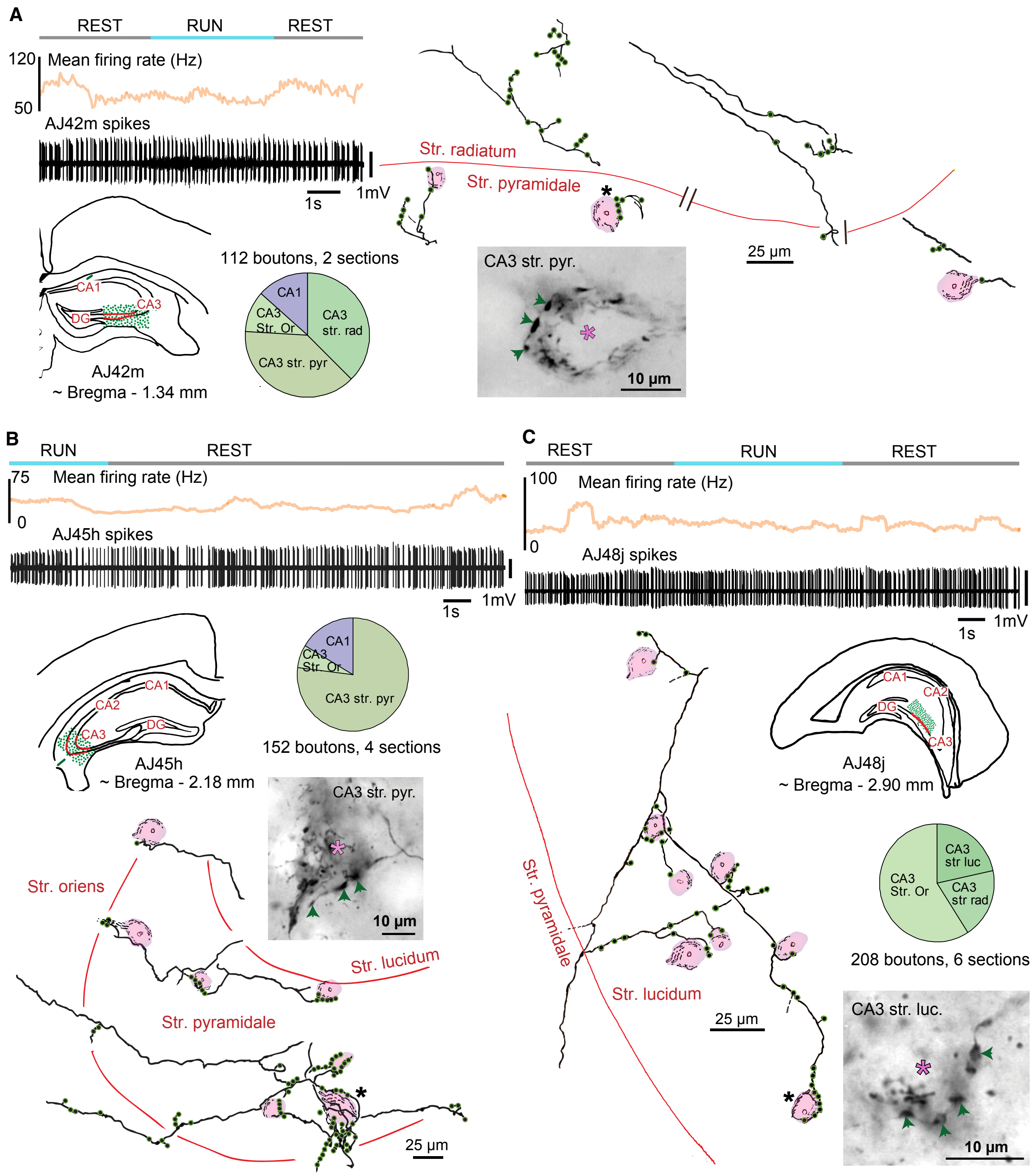

Figure 4. Teevra Neurons Innervate Interneurons in Spatially Restricted Domains of CA3

Teevra neurons were identified on the bases of not changing their mean firing rate during REST versus RUN and short burst duration during CA1 theta (upper panels). Reconstructions of axonal collaterals (green, boutons) of labeled cells reveal that Teevra neurons, AJ42m (A), AJ45h (B), and AJ48j (C) innervate interneurons in the CA3 region. Innervated interneuron somata (shaded pink) are identified by endogenous biotin in mitochondria. Pie charts show representative samples of bouton distribution in different areas and layers. Light micrographs of axonal varicosities (arrows) visualized by HRP enzyme reaction adjacent to cell bodies of individual interneurons (asterisks) rich in endogenous biotin in mitochondria (black in cytoplasm) as revealed by the color reaction.

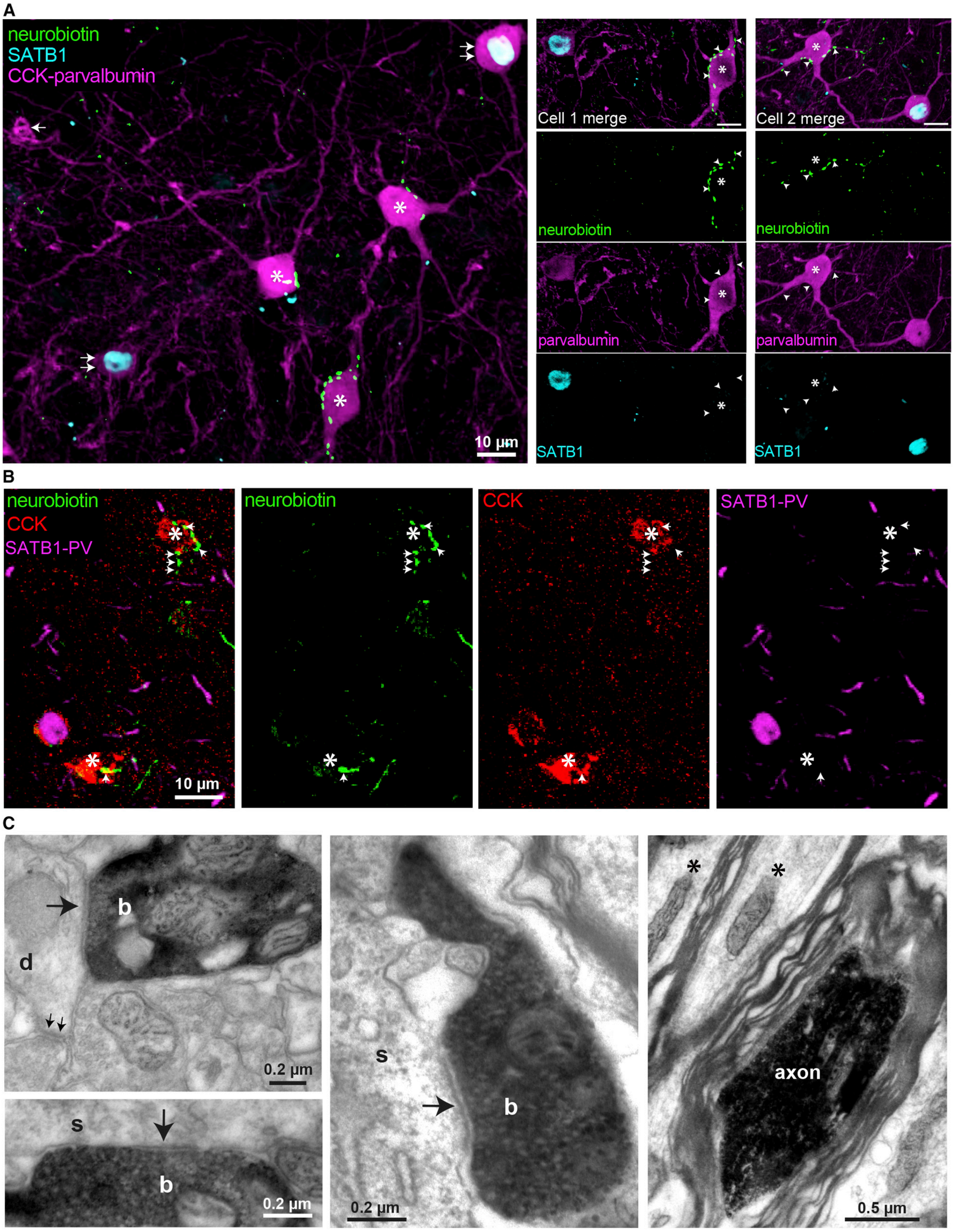

(legend on next page)

(Figures 5A and S2). Another Teevra neuron, MS90g, was more weakly labeled, and, although we followed the axon and branches both by fluorescence microscopy and HRP reactions, the terminal boutons were not well resolved in fluorescence microscopy. Triple immunoreactions for PV, SATB1, and CCK in immunofluorescence followed by HRP reaction for bouton visualization helped to identify 6 innervated somata by light microscopy. All targeted neurons were PV^+ and SATB1 immunonegative (Table S1). This combination is a strong indicator of axo-axonic cells in the CA1 and CA3 areas (Viney et al., 2013).

To demonstrate the remarkable cell-type selectivity of Teevra cells, we estimated the proportion of PV^+ neurons that could be classified as axo-axonic cells in the CA3 region of mouse hippocampus based on their molecular marker combination (30% of PV cells, Figure S3). This has allowed us to estimate the probability of the observed number of axo-axonic cells as synaptic targets, if all PV^+ cells were innervated uniformly. The probability for AJ42m (7 axo-axonic cell targets found sequentially) is $p = 0.3^7 = 2.1 \times 10^{-4}$, and for MS90g (6 axo-axonic cell targets found sequentially) is $p = 0.3^6 = 7.2 \times 10^{-4}$. Therefore, we conclude that Teevra cells selectively target axo-axonic among PV^+ neurons of the CA3 hippocampal area, but this does not exclude other interneuron types as we also found some CCK^+ interneurons (4 somata, AJ42m, Figure 5B) as target cells. These 4 CCK^+ targeted profiles were confirmed to be PV^-, $SATB1^-$, $somatostatin^-$ (SST), and $calbindin^-$ (CB). This combination of molecular markers is indicative of CCK basket cells (Lasztóczi et al., 2011) in CA3. Interestingly, both these neuronal populations fire spikes at the peak or ascending phase of dorsal CA1 pyramidal layer theta LFP oscillations (Somogyi et al., 2013), out of phase with the trough firing of MS Teevra cells.

Electron microscopic examination of the main axons of Teevra cells in the fimbria adjacent to the CA3 area showed that they are covered by myelin sheaths (AJ45h, Figure 5C), which are 2 to 3 times thicker than those of nearby axons of CA3 pyramidal cells. The main axonal branches in CA3 are also myelinated, and the terminal collaterals are unmyelinated and 0.1 to 0.3 μm thick forming boutons in clusters. We tested the probability of predicting synaptic junctions based on axonal swellings next to a target cell. All the boutons in a tested area (n = 11 boutons, AJ45h) formed type II synaptic junctions (Figure 5C) with two nearby interneuron somata in CA3b stratum pyramidale (n = 5 and 6 boutons per soma, respectively). The boutons of AJ42m tested by electron microscopy (n = 12) were distributed in CA3c strata pyramidale, lucidum, and radiatum. They formed synapses with an interneuron soma (n = 5 synapses, Figure 5C), 4 interneuron dendrites identified by receiving additional type I synapses (Figure 5C), and 3 unidentified dendritic shafts, which received no additional synapse in the range of sections that were followed.

Rhythmicity of Teevra Cells along the Septo-temporal Axis of the Hippocampus

Theta oscillations are traveling waves along the septo-temporal and medio-lateral extent of the hippocampal formation, and the power but not frequency of theta oscillations decreases along the longitudinal axis (Lubenov and Siapas, 2009; Patel et al., 2012; Long et al., 2015). Location of the somata of Teevra cells relative to the midline of the MS predicted the axonal distribution in the left or right hippocampus respectively as their axons did not cross the midline (Table 1). Teevra cells have multiple thick, long, and non-spiny dendrites originating from the soma. The dendrites branch infrequently in the septum and may cross the septal midline (Figure 6A). As Teevra cells innervated distinct and restricted domains of CA3 along the septo-temporal axis of the hippocampus, we asked whether the rhythmicity index, oscillatory frequency, and firing-phase preference of septo-CA3 projecting Teevra neurons showed any correlation with the innervated hippocampal area. Using 3D Euclidian distance along the hippocampal formation (see STAR Methods), we have observed that the rhythmicity of the firing of septal neurons decreases the more caudal the termination in the hippocampus (linear correlation coefficient: $r = -0.96$, $p = 0.0001$, $n = 8$ neurons, Figure 6B), but the oscillatory burst firing at theta frequency does not change ($p = 0.27$, $n = 8$ neurons, Figure 6B). This shows that the depth of modulation of rhythmic GABAergic input to CA3 from the MS decreases the more caudo-ventral the termination in CA3. We have also assessed the mean firing-phase distribution of neurons across the septo-temporal axis and found no correlation between these two variables (angular-linear correlation coefficient: $r = 0.38$, $p = 0.57$, $n = 8$ neurons).

DISCUSSION

We have demonstrated that a population of septal GABAergic neurons selectively target the CA3 region, which predicts that other regions of the hippocampus and related cortical areas also receive region and target cell-type-specific subcortical inputs. This organizational principle endows distinct cell types in the MS and diagonal band nuclei with a flexible role in coordinating functionally related cortical areas with each parallel pathway adapted to the specific role and requirements of its target area. Teevra cells formed the largest subpopulation of

Figure 5. Postsynaptic Targets of Teevra Neurons Are PV^+ or CCK^+ Interneurons

(A) Left, Axonal terminals (green) of Teevra cell AJ42m innervate PV^+ (magenta, asterisks) and SATB1-negative (cyan) cells in a basket-like formation also following their dendrites (merged channels). Nearby PV^+ and $SATB1^+$ cells (double arrows) and a CCK^+ cell (single arrow) were not innervated. CCK and PV were sequentially reacted and imaged. Right, two innervated cells from left panel channel by channel.

(B) Axonal terminals (green, arrows) of Teevra cell AJ42m innervate two interneurons (asterisks), which are CCK^+ (red) and $SATB1^-$ and PV^- (magenta, sequential reactions) in CA3 str. radiatum. Bottom left of image, non-targeted $SATB1^+$ PV-immunonegative neuron.

(C) Electron micrographs of Teevra cells AJ42m (left) and AJ45h (middle and right) in CA3. Boutons made type II synaptic junctions (single arrows) with a dendrite (d) and two different somata (s) of interneurons. The postsynaptic dendrite received a type I synapse (double arrows). Right, the main axon of AJ45h is heavily myelinated. Surrounding projection axons (asterisks) in the fimbria have thinner myelin sheaths. Imaging details (z-thickness in micrometers, z-projection type): (A) left, 34.48 μm SD projection; middle, 9.93 μm SD projection; right, 12.06 μm SD projection; (B) 4.00 μm, average intensity projection. See also Figures S2 and S3.

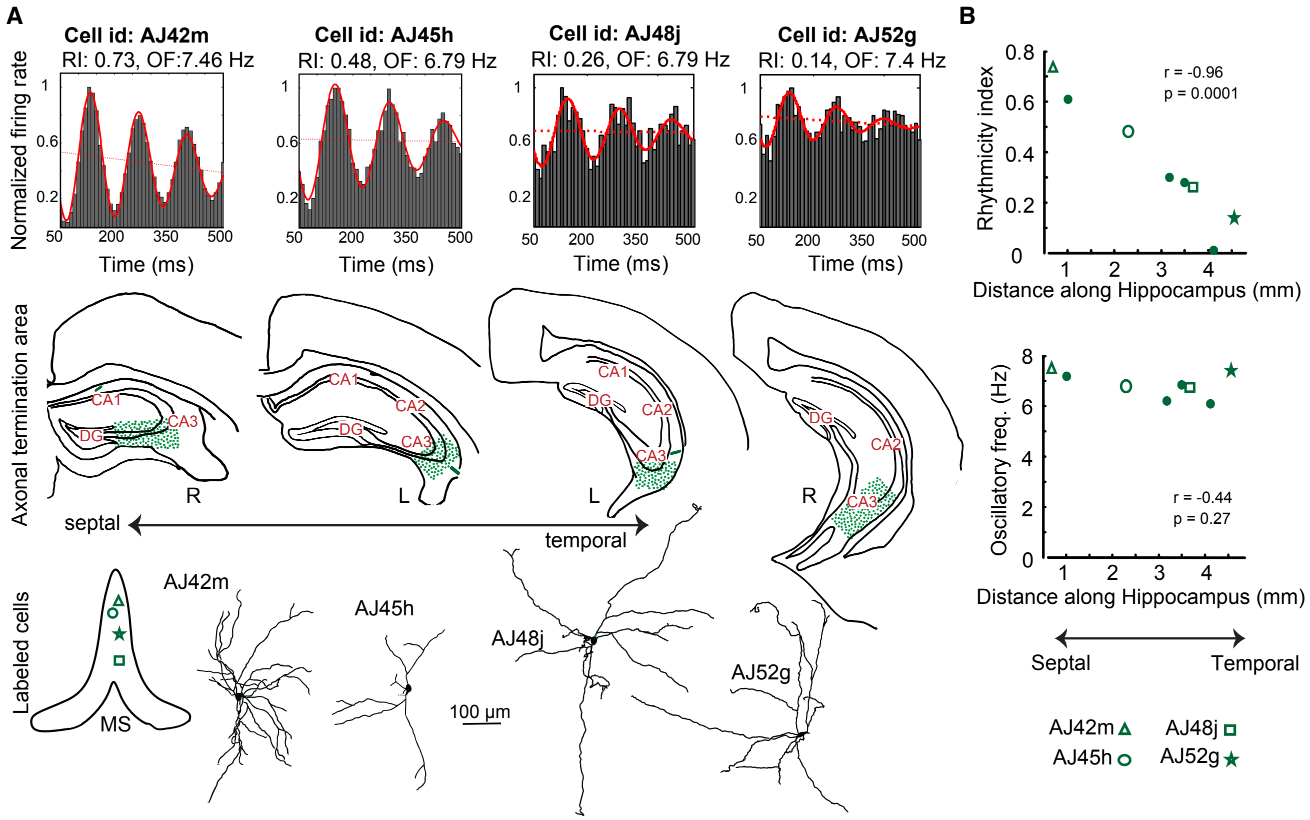

Figure 6. The Degree of Rhythmicity of Teevra Neurons Predicts Their Septo-temporal Termination Zone in the CA3

(A) Rhythmicity index (RI), oscillatory frequency (OF), and termination areas (green, dotted) in the CA3 area of labeled septal neurons showing that rhythmicity index decreases the more caudal the termination in CA3; outlines of the hippocampus (left, L; right, R) were rotated to display them in the same orientation. Bottom, position of cell bodies in the medial septum (projected onto one coronal plane) and reconstructions of their dendritic trees (AJ45h partial).

(B) Linear negative correlation between rhythmicity index (during RUN) and the septo-temporal termination of the axons along the hippocampus ($r = -0.96$, $p = 0.0001$) for all labeled Teevra neurons. There is no correlation between the oscillatory frequency of firing of the neurons during RUN and the termination of the axons ($p = 0.27$).

MS rhythmic neurons, which have been hypothesized to be the coordinators of hippocampal theta oscillations (Alonso et al., 1987; Gaztelu and Buño, 1982; Gogolák et al., 1968; Petsche et al., 1962; Stumpf et al., 1962). Teevra neurons have a short burst duration, do not significantly change their firing rate from REST to RUN, and fire action potentials at dorsal hippocampal CA1 troughs recorded in stratum pyramidale, coincident with the maximal firing of CA1 pyramidal cells (Mizuseki et al., 2009; Csicsvari et al., 1999).

We focused on neurons showing rhythmicity index of more than 0.1, and all such cells labeled and tested were GABAergic, but this does not exclude that less rhythmic GABAergic neurons also exist in the MS. Identified initially by their activity patterns, subsequent juxtacellular labeling of Teevra cells revealed their axonal termination area and synaptic target neurons. The most remarkable feature of Teevra cells is their selective termination in restricted spatial domains along septo-temporal axis of CA3, largely avoiding other hippocampal areas. These findings reveal an unexpected sophistication in the spatiotemporal organization of septo-hippocampal projection. Based on our analysis of the synaptic targets of Teevra cells, and assuming that the high-frequency bursts fired at the trough of the CA1 theta leads to inhibition, we propose that Teevra cells innervate those CA3 interneurons, such as axo-axonic cells and CCK basket cells (Lasztóczi et al., 2011; Somogyi et al., 2013), which preferentially fire around the peak of theta. This would lead to the disinhibition of CA3 pyramidal cell assemblies (Tóth et al., 1997), driving pyramidal cell firing in CA1 at the trough of theta in the pyramidal layer. Because axo-axonic cells do not innervate other interneurons, the coincidence of Teevra cell firing and the highest discharge probability of pyramidal cells also supports a disinhibitory role (Figure 7). Consistent with the proposed disinhibition of CA3 pyramidal cells by Teevra cells, pyramidal cells fire at the highest rate during the trough of CA1 theta oscillations in anesthetized rats (Lasztóczi et al., 2011). In this temporally coordinated circuit, during retrieval of stored contextual associations around the theta trough (Hasselmo et al., 2002), disinhibition provided by Teevra cells may enable the CA3 pyramidal cell output to contribute to temporal coding in the CA1 ensemble (Fernández-Ruiz et al., 2017; Middleton and McHugh, 2016). Such a proposed role remains to be tested directly.

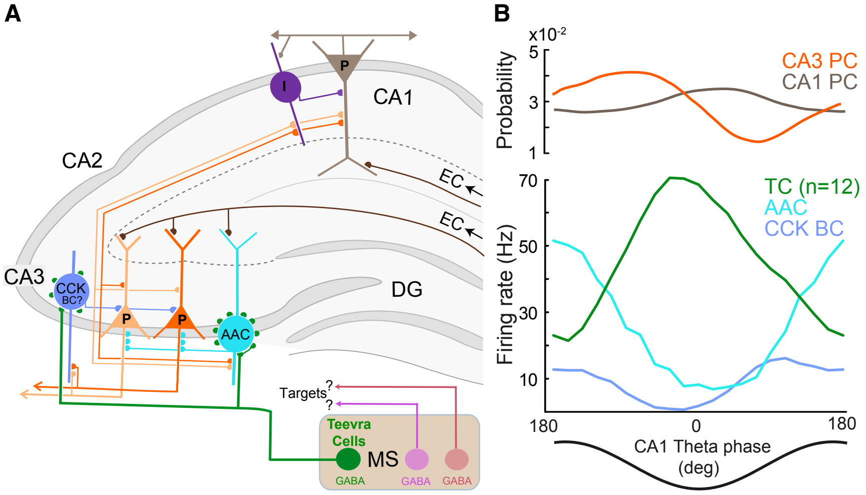

Figure 7. Theta Firing-Phase Selectivity and Schematic Summary of the Place of GABAergic MS Teevra Cells in the Hippocampal Circuit

(A) Teevra cells (green) preferentially innervate CA3 axo-axonic (AAC, cyan) and putative CCK-expressing basket cells (CCK, violet), which in turn target the axon initial segment and somata of CA3 pyramidal cells, respectively. The entorhinal cortical (EC) input to CA1 and CA3 innervates pyramidal cells and those GABAergic cells, such as AACs, which have dendrites in the termination zone. Pyramidal cells (P) provide recurrent input to interneurons and to other pyramidal cells and project to other cortical and subcortical areas (arrows). The termination area and target cell selectivity of other GABAergic MS neurons (magenta, brown, and others) remain to be determined. One general GABAergic neuron is shown in CA1 (I, lilac). Dentate granule cells, other types of GABAergic interneurons, and cholinergic and glutamatergic MS cells are not shown.

(B) Theta-phase relationships of neurons shown in (A) from recorded data, referenced to dorsal CA1 pyramidal cell layer LFP. On average, Teevra cells (n = 12, current study) discharge maximally at the trough of CA1 theta oscillations inhibiting AACs (data from Viney et al., 2013, non-anesthetized rat, CA1 and CA2 AACs averaged) and putative CCK basket cells (Lasztóczi et al., 2011, anesthetized rat, CA3) leading to disinhibition of CA3 pyramidal cells, which provide the largest excitatory input to CA1 pyramidal cells (average firing probabilities from Mizuseki et al., 2009). The cell-type-specific temporal modulation of firing rates during theta cycles contributes to the implementation of oscillatory increases and decreases of excitability in pyramidal cell networks via subcellular compartment specific disinhibition.

Synaptic Targets of Theta Synchronized Teevra Cells in CA3

Following the discovery that septal GABAergic neurons selectively innervate hippocampal interneurons (Freund and Antal, 1988), it was hypothesized that PV^+ septal neurons firing at the peak of CA1 theta inhibit both trough firing MS neurons and GABAergic hippocampal neurons that innervated pyramidal cell dendrites in CA1; in turn, theta trough-preferring MS neurons innervate both the peak firing MS neurons and "peri-somatic" terminating inhibitory cells in CA1 (Borhegyi et al., 2004). Indeed, some trough-preferring Teevra cells innervate other PV^+ MS neurons, but the firing phase of these target cells is unknown. Moreover, none of the theta trough-firing rhythmic Teevra cells innervated the CA1 significantly and instead targeted PV^+ axo-axonic cells and CCK^+ cells in the CA3. The binary phase preference hypothesis is also complicated by the fact that both hippocampal GABAergic cells (Klausberger and Somogyi 2008) and theta rhythmically firing GABAergic MS neurons fire at all phases of CA1 theta. The key missing information for explaining this diversity has been the axonal area and target cell preference of any septal neuron with known theta-phase firing. The Teevra cells reported here show one example of a sophisticated and highly selective septo-hippocampal connection.

Information on the firing-phase preference of identified CA3 interneurons is sparse in awake animals. In anaesthetized rats, identified axo-axonic cells in CA3 fired bursts of spikes at the peak of dorsal CA1 theta oscillations (Varga et al., 2014; Viney et al., 2013). Axo-axonic cells innervate exclusively the axon initial segment of pyramidal neurons, which is particularly well developed in CA3 with up to 150 synapses on a single-axon initial segment (Kosaka, 1980). Their action is mediated by $GABA_A$ receptors (Buhl et al., 1994) with fast (~1.7 ms) inhibitory postsynaptic currents in their synaptic targets (Ganter et al., 2004), at the site where the action potential is generated. Interneurons expressing CCK also fire spikes at around the peak of CA1 theta oscillations (Lasztóczi et al., 2011). Thus, the axo-axonic and CCK target cells, postsynaptic to Teevra neurons, fire preferentially at the theta peak, counter-phased with the rhythmic input from Teevra cells at the trough of theta. This counter-phase firing has been suggested earlier for CA1 (Somogyi et al., 2013) and might indeed be a biological mechanism of theta-phase modulation of long-range synaptic partners. However, theta peak firing MS neurons with long burst duration had no terminals in the CA1 or CA3, but instead their main axon projected beyond the hippocampus likely innervating extrahippocampal structures. Our results do not exclude the innervation of various other interneuron types by Teevra cells in CA3 such as PV^+ basket cells, which fire phase locked to the trough of CA1 theta (Tukker et al., 2013), but coincident firing with and synaptic input from Teevra cells is unlikely. It is possible that other MS GABAergic neuronal types with theta-phase preference different from Teevra cells also innervate CA3. Besides a dominant role of septal GABAergic neurons in determining interneuronal firing-phase preference, interaction with other rhythmic synaptic inputs, e.g., from the raphe nuclei, supramammillary nucleus, and from local interneurons and pyramidal cells, may also contribute to the determination of theta-phase firing preference of interneurons. The effects of a potential synaptic influence of CCK-expressing interneurons, which are innervated by Teevra cell, on axo-axonic cells, similar to PV^+ basket cells innervated by CCK^+ interneurons in CA1 (Karson et al., 2009) remain to be tested.

Synaptic inputs to CA1 are temporally organized within theta and gamma timescales. The CA3 pyramidal cell input at the

trough and descending phase of CA1 pyramidal layer theta coincides with slow gamma (30–80 Hz) oscillations, while the medium gamma (60–120 Hz) is coupled to the peak of pyramidale theta oscillations (Fernández-Ruiz et al., 2017; Lasztóczi and Klausberger, 2014; Schomburg et al., 2014). It was previously suggested that MS neuronal firing at gamma intraburst frequencies (30–120 Hz) might contribute to these oscillations (Borhegyi et al., 2004; Viney et al., 2013). However, two oscillations occurring at the same frequency might not lead to amplification unless they are phase coupled. Unlike under anesthesia, MS Teevra neurons in awake animals, on average, had higher intraburst frequencies than medium gamma (up to 300 Hz during RUN). The phase coupling of Teevra cell spikes to various gamma oscillations, especially putative CA3 coordinated slow gamma, remains to be investigated with multielectrode arrays to reveal current sources.

Teevra Cell Firing Is Modulated over Multiple Timescales: Within Theta Cycles and during Rest and Running

A fascinating feature of firing of Teevra cells is that the firing rate did not change from rest to running significantly or could even decrease during RUN. This is in contrast to the firing of most CA1 interneurons, which increased their firing rate from rest to running periods (Czurkó et al., 2011; Varga et al., 2012) possibly due to a combination of decreased inhibition from MS GABAergic cells, increased excitatory input from CA3 pyramidal cells and/or long range inputs (Fuhrmann et al., 2015). Although the firing rate of Teevra cells may not be different during REST and RUN periods, the temporal dynamics of firing pattern changed with a clear increase in the rhythmicity index and oscillatory frequency during running. If on average each action potential provides a similar amount of GABA released at the synaptic terminals, why do Teevra cells maintain a high level of GABA release during rest when their targets are much less theta rhythmic? We have shown that Teevra cells continue to fire at the negative deflections of slow irregular activity, increasing their firing around the hippocampal LFP troughs, irrespective of the frequency at which these occur, mirroring the heightened excitation in CA1.

Sharp wave ripple episodes are more frequent during rest and consummatory behavior representing the highest population activity in the hippocampus. During sharp wave ripples, some MS GABAergic neurons are strongly active (Borhegyi et al., 2004; Viney et al., 2013) while others are inhibited (Borhegyi et al., 2004; Dragoi et al., 1999) or do not change their firing like Teevra cells. MS GABAergic cells are innervated by hippocampo-septal GABAergic projection neurons (Tóth et al., 1993), which are strongly activated during sharp wave ripple events (Jinno et al., 2007; Katona et al., 2017). These MS cells might correspond to the sharp wave ripple inhibited rhythmic population (Dragoi et al., 1999). Teevra cells on the other hand did not change their firing rate during sharp wave ripples suggesting that these are not targets of hippocampo-septal projection neurons. Thus, during REST periods, Teevra cells differentiated between sharp wave ripples and the less regular duration increased hippocampal excitability events, when their firing was strongly coupled to hippocampal LFP troughs.

Teevra Cells Spatial Organization and Traveling Theta Waves

The power of theta oscillations decreases along the longitudinal hippocampal axis and the phase of theta shifts by 180° across the septo-temporal extent of the hippocampus (Lubenov and Siapas, 2009; Patel et al., 2012). Strikingly, we have found a strong correlation of the rhythmicity of Teevra neurons along the longitudinal axis. Highly rhythmic septal Teevra neurons innervate interneurons in the septal pole and less rhythmic neurons innervate interneurons in the temporal pole of the hippocampus, while the oscillatory frequency at which MS input is organized does not change along this axis. This parallels the decrease in theta power and reduction in theta rhythmic neurons in ventral compared to dorsal CA3 (Royer et al., 2010).

Multiple mechanisms have been suggested for the generation of theta waves. One of the suggestions was the existence of a chain of oscillators residing within the septal area and theta waves would be a reflection of the phase delayed septal outputs (Patel et al., 2012). The septo-temporally restricted axons demonstrated here could support mechanisms of locally variable theta oscillations, as suggested by Kang et al. (2015). However, we have found no correlation between the firing-phase preference and position of the axon.

The axons of septal GABAergic neurons are heavily myelinated (Borhegyi et al., 2004), which we confirmed here, and exhibit high conduction velocities (0.5–5 m/s, Jones et al., 1999). Thus, the conduction delay is unlikely to contribute to a significant delay of transmission as it is an order of magnitude faster than the propagation velocity (0.16 m/s, Patel et al., 2012) of theta waves. However, delays of similar theta wave velocity were reported in hippocampal CA3 slices *in vitro* (Miles et al., 1988) while blocking glutamatergic transmission; thus, the hippocampal system is capable of generating such a wave through inhibitory connections. *In vivo*, the uniquely positioned Teevra neurons might provide a temporally coherent synchronized GABAergic input, which rhythmically inhibits CA3 interneurons along the hippocampal long axis, thus coordinating pyramidal cell excitability.

Outlook

We have defined a novel septo-hippocampal GABAergic cell type using congruent neuronal features including physiological parameters, molecular expression profiles, and axonal termination area. The results demonstrate the cellular diversity in the MS and provide a spatiotemporal framework for understanding the long-range, parallel subcortical innervation, which coordinates network oscillations in the cortex via local inhibitory neurons. We hypothesize that such cortical region-specific GABAergic innervation by physiologically distinct septal neuronal types supports the coordination of network oscillations.

STAR★METHODS

Detailed methods are provided in the online version of this paper and include the following:

- KEY RESOURCES TABLE
- CONTACT FOR REAGENT AND RESOURCE SHARING

- EXPERIMENTAL MODEL AND SUBJECT DETAILS
- METHOD DETAILS
  - Surgery
  - Behavioral procedures
  - Recordings and single unit identification
  - Juxtacellular labeling
  - Tissue processing and immunohistochemistry
  - Anatomical data analysis
  - Calculation of 3D distance in the hippocampus
  - Electrophysiological data analysis
  - Hierarchical clustering
- QUANTIFICATION AND STATISTICAL ANALYSIS
- DATA AND SOFTWARE AVAILABILITY

SUPPLEMENTAL INFORMATION

Supplemental Information includes three figures, one table, and two movies and can be found with this article online at https://doi.org/10.1016/j.neuron.2017.10.033.

A video abstract is available at https://doi.org/10.1016/j.neuron.2017.10.033#mmc5.

AUTHOR CONTRIBUTIONS

Conceptualization, A.J. and P.S.; Methodology, A.J., M.S., T.J.V., D.D., and P.S.; Investigation, A.J., M.S., and P.S.; Writing – Original Draft, A.J.; Writing – Review & Editing, M.S., T.J.V., D.D., and P.S.; Funding Acquisition, D.D. and P.S.; Supervision, D.D., P.S., and T.J.V.

ACKNOWLEDGMENTS

We would like to thank Linda Katona for training and critical advice during the course of the project. We thank Rob Stewart and Linda Katona for providing scripts for rhythmicity and sharp wave ripple analysis. We thank Thomas Klausberger and Balint Lasztóczi for training and advice on analyzing the slow irregular activity. We thank Vitor Lopes dos Santos, Balazs Hangya, Stephanie Trouche, and John Tukker for comments on an earlier version of the manuscript. We thank Michael Howarth for 3D reconstruction of AJ48j and Amar Sharma for somato-dendritic reconstructions. We thank Kristina Wagner, Ben Micklem, and Katja Hartwich for excellent technical assistance and Joszef Somogyi for advice on confocal microscopy. A.J. was funded by Felix scholarship, Oxford. The virtual reality setup was funded to D.D. by the Medical Research Foundation (award C0443). The research was supported by the Medical Research Council UK (awards MC_UU_12024/4 and MC_UU_12024/3) and the Wellcome Trust (award 108726/Z/15/Z).

Received: June 19, 2017
Revised: August 23, 2017
Accepted: October 25, 2017
Published: November 30, 2017

REFERENCES

Alonso, A., Gaztelu, J.M., Buño, W., Jr., and García-Austt, E. (1987). Cross-correlation analysis of septohippocampal neurons during theta-rhythm. Brain Res. *413*, 135–146.

Amaral, D.G., and Witter, M.P. (1989). The three-dimensional organization of the hippocampal formation: A review of anatomical data. Neuroscience *31*, 571–591.

Bakker, R., Tiesinga, P., and Kötter, R. (2015). The Scalable Brain Atlas: Instant web-based access to public brain atlases and related content. Neuroinformatics *13*, 353–366.

Berry, S.D., and Thompson, R.F. (1979). Medial septal lesions retard classical conditioning of the nicitating membrane response in rabbits. Science *205*, 209–211.

Borhegyi, Z., Varga, V., Szilágyi, N., Fabo, D., and Freund, T.F. (2004). Phase segregation of medial septal GABAergic neurons during hippocampal theta activity. J. Neurosci. *24*, 8470–8479.

Brun, V.H., Leutgeb, S., Wu, H.Q., Schwarcz, R., Witter, M.P., Moser, E.I., and Moser, M.-B. (2008). Impaired spatial representation in CA1 after lesion of direct input from entorhinal cortex. Neuron *57*, 290–302.

Buhl, E.H., Han, Z.S., Lörinczi, Z., Stezhka, V.V., Karnup, S.V., and Somogyi, P. (1994). Physiological properties of anatomically identified axo-axonic cells in the rat hippocampus. J. Neurophysiol. *71*, 1289–1307.

Buzsáki, G. (1984). Feed-forward inhibition in the hippocampal formation. Prog. Neurobiol. *22*, 131–153.

Calandreau, L., Jaffard, R., and Desmedt, A. (2007). Dissociated roles for the lateral and medial septum in elemental and contextual fear conditioning. Learn. Mem. *14*, 422–429.

Csicsvari, J., Hirase, H., Czurkó, A., Mamiya, A., and Buzsáki, G. (1999). Oscillatory coupling of hippocampal pyramidal cells and interneurons in the behaving Rat. J. Neurosci. *19*, 274–287.

Czurkó, A., Huxter, J., Li, Y., Hangya, B., and Muller, R.U. (2011). Theta phase classification of interneurons in the hippocampal formation of freely moving rats. J. Neurosci. *31*, 2938–2947.

Dragoi, G., Carpi, D., Recce, M., Csicsvari, J., and Buzsáki, G. (1999). Interactions between hippocampus and medial septum during sharp waves and theta oscillation in the behaving rat. J. Neurosci. *19*, 6191–6199.

Fernández-Ruiz, A., Oliva, A., Nagy, G.A., Maurer, A.P., Berényi, A., and Buzsáki, G. (2017). Entorhinal-CA3 dual-input control of spike timing in the hippocampus by theta-gamma coupling. Neuron *93*, 1213–1226.e5.

Ferraguti, F., Cobden, P., Pollard, M., Cope, D., Shigemoto, R., Watanabe, M., and Somogyi, P. (2004). Immunolocalization of metabotropic glutamate receptor 1α (mGluR1α) in distinct classes of interneuron in the CA1 region of the rat hippocampus. Hippocampus *14*, 193–215.

Foster, D.J., and Wilson, M.A. (2007). Hippocampal theta sequences. Hippocampus *17*, 1093–1099.

Freund, T.F., and Antal, M. (1988). GABA-containing neurons in the septum control inhibitory interneurons in the hippocampus. Nature *336*, 170–173.

Fuhrmann, F., Justus, D., Sosulina, L., Kaneko, H., Beutel, T., Friedrichs, D., Schoch, S., Schwarz, M.K., Fuhrmann, M., and Remy, S. (2015). Locomotion, theta oscillations, and the speed-correlated firing of hippocampal neurons are controlled by a medial septal glutamatergic circuit. Neuron *86*, 1253–1264.

Ganter, P., Szücs, P., Paulsen, O., and Somogyi, P. (2004). Properties of horizontal axo-axonic cells in stratum oriens of the hippocampal CA1 area of rats in vitro. Hippocampus *14*, 232–243.

Gaztelu, J.M., and Buño, W., Jr. (1982). Septo-hippocampal relationships during EEG theta rhythm. Electroencephalogr. Clin. Neurophysiol. *54*, 375–387.

Gielow, M.R., and Zaborszky, L. (2017). The Input-output relationship of the cholinergic basal forebrain. Cell Rep. *18*, 1817–1830.

Gogolák, G., Stumpf, C., Petsche, H., and Sterc, J. (1968). The firing pattern of septal neurons and the form of the hippocampal theta wave. Brain Res. *7*, 201–207.

Gray, E.G. (1959). Axo-somatic and axo-dendritic synapses of the cerebral cortex: An electron microscope study. J. Anat. *93*, 420–433.

Harris, K.D., Henze, D.A., Czicvari, J., Hirase, H., and Buzsaki, G. (2000). Accuracy of tetrode spike separation as determined by simultaneous intracellular and extracellular measurements. J. Neurophysiol. *84*, 401–414.

Hasselmo, M.E., Bodelón, C., and Wyble, B.P. (2002). A proposed function for hippocampal theta rhythm: Separate phases of encoding and retrieval enhance reversal of prior learning. Neural Comput. *14*, 793–817.

Huh, C.Y.L., Goutagny, R., and Williams, S. (2010). Glutamatergic neurons of the mouse medial septum and diagonal band of Broca synaptically drive hippocampal pyramidal cells: Relevance for hippocampal theta rhythm. J. Neurosci. *30*, 15951–15961.

Jinno, S., Klausberger, T., Marton, L.F., Dalezios, Y., Roberts, J.D., Fuentealba, P., Bushong, E.A., Henze, D., Buzsáki, G., and Somogyi, P.

(2007). Neuronal diversity in GABAergic long-range projections from the hippocampus. J. Neurosci. *27*, 8790–8804.

Jones, G.A., Norris, S.K., and Henderson, Z. (1999). Conduction velocities and membrane properties of different classes of rat septohippocampal neurons recorded in vitro. J. Physiol. *517*, 867–877.

Justus, D., Dalügge, D., Bothe, S., Fuhrmann, F., Hannes, C., Kaneko, H., Friedrichs, D., Sosulina, L., Schwarz, I., Elliott, D.A., et al. (2017). Glutamatergic synaptic integration of locomotion speed via septoentorhinal projections. Nat. Neurosci. *20*, 16–19.

Kadir, S.N., Goodman, D.F.M., and Harris, K.D. (2014). High-dimensional cluster analysis with the masked EM algorithm. Neural Comput. *26*, 2379–2394.

Kaifosh, P., Lovett-Barron, M., Turi, G.F., Reardon, T.R., and Losonczy, A. (2013). Septo-hippocampal GABAergic signaling across multiple modalities in awake mice. Nat. Neurosci. *16*, 1182–1184.

Kang, D., Ding, M., Topchiy, I., Shifflett, L., and Kocsis, B. (2015). Theta-rhythmic drive between medial septum and hippocampus in slow-wave sleep and microarousal: A Granger causality analysis. J. Neurophysiol. *114*, 2797–2803.

Karson, M.A., Tang, A.-H., Milner, T.A., and Alger, B.E. (2009). Synaptic cross talk between perisomatic-targeting interneuron classes expressing cholecystokinin and parvalbumin in hippocampus. J. Neurosci. *29*, 4140–4154.

Katona, L., Lapray, D., Viney, T.J., Oulhaj, A., Borhegyi, Z., Micklem, B.R., Klausberger, T., and Somogyi, P. (2014). Sleep and movement differentiates actions of two types of somatostatin-expressing GABAergic interneuron in rat hippocampus. Neuron *82*, 872–886.

Katona, L., Micklem, B., Borhegyi, Z., Swiejkowski, D.A., Valenti, O., Viney, T.J., Kotzadimitriou, D., Klausberger, T., and Somogyi, P. (2017). Behavior-dependent activity patterns of GABAergic long-range projecting neurons in the rat hippocampus. Hippocampus *27*, 359–377.

King, C., Recce, M., and O'Keefe, J. (1998). The rhythmicity of cells of the medial septum/diagonal band of Broca in the awake freely moving rat: Relationships with behaviour and hippocampal theta. Eur. J. Neurosci. *10*, 464–477.

Klausberger, T., and Somogyi, P. (2008). Neuronal diversity and temporal dynamics: The unity of hippocampal circuit operations. Science *321*, 53–57.

Kosaka, T. (1980). The axon initial segment as a synaptic site: Ultrastructure and synaptology of the initial segment of the pyramidal cell in the rat hippocampus (CA3 region). J. Neurocytol. *9*, 861–882.

Lardi-Studler, B., Smolinsky, B., Petitjean, C.M., Koenig, F., Sidler, C., Meier, J.C., Fritschy, J.M., and Schwarz, G. (2007). Vertebrate-specific sequences in the gephyrin E-domain regulate cytosolic aggregation and postsynaptic clustering. J. Cell Sci. *120*, 1371–1382.

Lasztóczi, B., and Klausberger, T. (2014). Layer-specific GABAergic control of distinct gamma oscillations in the CA1 hippocampus. Neuron *81*, 1126–1139.

Lasztóczi, B., Tukker, J.J., Somogyi, P., and Klausberger, T. (2011). Terminal field and firing selectivity of cholecystokinin-expressing interneurons in the hippocampal CA3 area. J. Neurosci. *31*, 18073–18093.

Leão, R.N., Mikulovic, S., Leão, K.E., Munguba, H., Gezelius, H., Enjin, A., Patra, K., Eriksson, A., Loew, L.M., Tort, A.B., and Kullander, K. (2012). OLM interneurons differentially modulate CA3 and entorhinal inputs to hippocampal CA1 neurons. Nat. Neurosci. *15*, 1524–1530.

Leão, R.N., Targino, Z.H., Colom, L.V., and Fisahn, A. (2015). Interconnection and synchronization of neuronal populations in the mouse medial septum/diagonal band of Broca. J. Neurophysiol. *113*, 971–980.

Long, L.L., Bunce, J.G., and Chrobak, J.J. (2015). Theta variation and spatiotemporal scaling along the septotemporal axis of the hippocampus. Front. Syst. Neurosci. *9*, 37.

Lubenov, E.V., and Siapas, A.G. (2009). Hippocampal theta oscillations are travelling waves. Nature *459*, 534–539.

Middleton, S.J., and McHugh, T.J. (2016). Silencing CA3 disrupts temporal coding in the CA1 ensemble. Nat. Neurosci. *19*, 945–951.

Miles, R., Traub, R.D., and Wong, R.K. (1988). Spread of synchronous firing in longitudinal slices from the CA3 region of the hippocampus. J. Neurophysiol. *60*, 1481–1496.

Mizuseki, K., Sirota, A., Pastalkova, E., and Buzsáki, G. (2009). Theta oscillations provide temporal windows for local circuit computation in the entorhinal-hippocampal loop. Neuron *64*, 267–280.

Morino, P., Herrera-Marschitz, M., Castel, M.N., Ungerstedt, U., Varro, A., Dockray, G., and Hökfelt, T. (1994). Cholecystokinin in Cortico-striatal Neurons in the Rat: Immunohistochemical Studies at the Light and Electron Microscopical Level. Eur. J. Neurosci. *6*, 681–692.

Neunuebel, J.P., and Knierim, J.J. (2014). CA3 retrieves coherent representations from degraded input: Direct evidence for CA3 pattern completion and dentate gyrus pattern separation. Neuron *81*, 416–427.

Patel, J., Fujisawa, S., Berényi, A., Royer, S., and Buzsáki, G. (2012). Traveling theta waves along the entire septotemporal axis of the hippocampus. Neuron *75*, 410–417.

Petsche, H., Stumpf, C., and Gogolak, G. (1962). [The significance of the rabbit's septum as a relay station between the midbrain and the hippocampus. I. The control of hippocampus arousal activity by the septum cells]. Electroencephalogr. Clin. Neurophysiol. *14*, 202–211.

Pinault, D. (1996). A novel single-cell staining procedure performed in vivo under electrophysiological control: Morpho-functional features of juxtacellularly labeled thalamic cells and other central neurons with biocytin or Neurobiotin. J. Neurosci. Methods *65*, 113–136.

Robinson, J., Manseau, F., Ducharme, G., Amilhon, B., Vigneault, E., El Mestikawy, S., and Williams, S. (2016). Optogenetic activation of septal glutamatergic neurons drive hippocampal theta rhythms. J. Neurosci. *36*, 3016–3023.

Royer, S., Sirota, A., Patel, J., and Buzsáki, G. (2010). Distinct representations and theta dynamics in dorsal and ventral hippocampus. J. Neurosci. *30*, 1777–1787.

Schindelin, J., Arganda-Carreras, I., Frise, E., Kaynig, V., Longair, M., Pietzsch, T., Preibisch, S., Rueden, C., Saalfeld, S., Schmid, B., et al. (2012). Fiji: An open-source platform for biological-image analysis. Nat. Methods *9*, 676–682.

Schomburg, E.W., Fernández-Ruiz, A., Mizuseki, K., Berényi, A., Anastassiou, C.A., Koch, C., and Buzsáki, G. (2014). Theta phase segregation of input-specific gamma patterns in entorhinal-hippocampal networks. Neuron *84*, 470–485.

Simon, A.P., Poindessous-Jazat, F., Dutar, P., Epelbaum, J., and Bassant, M.-H. (2006). Firing properties of anatomically identified neurons in the medial septum of anesthetized and unanesthetized restrained rats. J. Neurosci. *26*, 9038–9046.

Sławińska, U., and Kasicki, S. (1998). The frequency of rat's hippocampal theta rhythm is related to the speed of locomotion. Brain Res. *796*, 327–331.

Sloviter, R.S., and Nilaver, G. (1987). Immunocytochemical localization of GABA-, cholecystokinin-, vasoactive intestinal polypeptide-, and somatostatin-like immunoreactivity in the area dentata and hippocampus of the rat. The Journal of Comparative Neurology *256*, 42–60.

Somogyi, P., Katona, L., Klausberger, T., Lasztóczi, B., and Viney, T.J. (2013). Temporal redistribution of inhibition over neuronal subcellular domains underlies state-dependent rhythmic change of excitability in the hippocampus. Philos. Trans. R. Soc. Lond. B Biol. Sci. *369*. Published online December 23, 2013. https://doi.org/10.1098/rstb.2012.0518.

Stumpf, C., Petsche, H., and Gogolak, G. (1962). The significance of the rabbit's septum as a relay station between the midbrain and the hippocampus. II. The differential influence of drugs upon both the septal cell firing pattern and the hippocampus theta activity. Electroencephalogr. Clin. Neurophysiol. *14*, 212–219.

Tóth, K., Borhegyi, Z., and Freund, T.F. (1993). Postsynaptic targets of GABAergic hippocampal neurons in the medial septum-diagonal band of broca complex. J. Neurosci. *13*, 3712–3724.

Tóth, K., Freund, T.F., and Miles, R. (1997). Disinhibition of rat hippocampal pyramidal cells by GABAergic afferents from the septum. J. Physiol. *500*, 463–474.

Tukker, J.J., Lasztóczi, B., Katona, L., Roberts, J.D., Pissadaki, E.K., Dalezios, Y., Márton, L., Zhang, L., Klausberger, T., and Somogyi, P. (2013). Distinct dendritic arborization and in vivo firing patterns of parvalbumin-expressing basket cells in the hippocampal area CA3. J. Neurosci. *33*, 6809–6825.

Unal, G., Joshi, A., Viney, T.J., Kis, V., and Somogyi, P. (2015). Synaptic Targets of medial septal projections in the hippocampus and extrahippocampal cortices of the mouse. J. Neurosci. *35*, 15812–15826.

Varga, C., Golshani, P., and Soltesz, I. (2012). Frequency-invariant temporal ordering of interneuronal discharges during hippocampal oscillations in awake mice. Proc. Natl. Acad. Sci. USA *109*, E2726–E2734.

Varga, C., Oijala, M., Lish, J., Szabo, G.G., Bezaire, M., Marchionni, I., Golshani, P., and Soltesz, I. (2014). Functional fission of parvalbumin interneuron classes during fast network events. eLife *3*. Published online November 6, 2014. https://doi.org/10.7554/eLife.04006.

Vertes, R.P., and Kocsis, B. (1997). Brainstem-diencephalo-septohippocampal systems controlling the theta rhythm of the hippocampus. Neuroscience *81*, 893–926.

Viney, T.J., Lasztoczi, B., Katona, L., Crump, M.G., Tukker, J.J., Klausberger, T., and Somogyi, P. (2013). Network state-dependent inhibition of identified hippocampal CA3 axo-axonic cells in vivo. Nat. Neurosci. *16*, 1802–1811.

Winson, J. (1978). Loss of hippocampal theta rhythm results in spatial memory deficit in the rat. Science *201*, 160–163.

Witter, M.P. (2007). Intrinsic and extrinsic wiring of CA3: Indications for connectional heterogeneity. Learn. Mem. *14*, 705–713.

Witter, M.P., Griffioen, A.W., Jorritsma-Byham, B., and Krijnen, J.L.M. (1988). Entorhinal projections to the hippocampal CA1 region in the rat: An underestimated pathway. Neurosci. Lett. *85*, 193–198.

Zar, J.H. (1999). Biostatistical Analysis, Fourth Edition (Prentice-Hall, Inc.).

STAR★METHODS

KEY RESOURCES TABLE

REAGENT or RESOURCE	SOURCE	IDENTIFIER
**Antibodies**		
Calbindin (CB) [host Rb; Dil 1:5000]	Swant	Cat. No. CB 38; RRID:AB_10000340
Calretinin (CR) [host Rb; Dil 1:500]	Swant	Cat. No. 7699/3H; RRID:AB_10000321
Metabotropic glutamate receptor 1a (mGluR1a) [host Gp; Dil 1:1000]	Frontier Institute	Cat. No. mGluR1a-GP-Af660; RRID:AB_2531897
Parvalbumin (PV) [host Rb; Dil 1:1000]	Swant	Cat. No. PV 28; RRID:AB_2315235
PV [host Gt; Dil 1:1000]	Swant	Cat. No. PVG 214; RRID:AB_10000345
PV [host Gp; Dil 1:5000]	Synaptic Systems	Cat. No. 195 004; RRID:AB_2156476
Pro-cholecystokinin (pro-CCK) [host Rb; Dil 1:500]	April 2005 gift (similar to Frontier Institute, CCK-pro-Rb-Af350)	Similar labeling to two non-commercial antibodies characterized by Sloviter and Nilaver (1987); Morino et al. (1994)
SATB1 (N-14) [host Rb; Dil 1:200]	Abcam	Cat. No. ab70004; RRID:AB_1270545
SATB1 (N-14) [host Gt; Dil 1:200]	Santa Cruz	Cat. No. sc-5989; RRID:AB_2184337
Somatostatin (SOM) [host Ms; Dil 1:500]	GeneTex	Cat. No. GTX71935; RRID:AB_383280
Somatostatin (SOM) [host Rb; Dil 1:50]	GenWay Biotech	Cat. No. 18-783-76392; RRID:AB_1027453
Vesicular GABA transporter (VGAT) [host Rb; Dil 1:500]	Synaptic Systems	Cat. No. 131 003; RRID:AB_887869
VGAT [host Gp; Dil 1:500]	Synaptic Systems	Cat. No. 131 004; RRID:AB_887873
Vesicular Glutamate transporter (VGlut2) [host Gp; Dil 1:2000]	Synaptic Systems	Cat. No. 135 404; RRID:AB_887884
**Chemicals, Peptides, and Recombinant Proteins**		
Neurobiotin Tracer	Vector Laboratories	Cat. No. SP-1120
**Critical Commercial Assays**		
Vectastain ABC kit	Vector Laboratories	Cat. No. 6100
VECTASHIELD Antifade Mounting Medium	Vector Laboratories	Cat. No. H-1000
**Experimental Models: Organisms/Strains**		
C57BL/6J	Charles River	JAX Mice Stock Number 000664
**Software and Algorithms**		
MATLAB	MathWorks	Version R2014a; https://uk.mathworks.com/products/matlab.html
Spike2	Cambridge Electronic Design Limited (CED)	Spike2 version 7 and version 8; http://ced.co.uk/
KlustaKwik	Harris et al., 2000	http://klustakwik.sourceforge.net/
Zen	Zeiss	Version 2.1; https://www.zeiss.co.uk/corporate/home.html
Fiji	ImageJ	Schindelin et al., 2012
Scalable Brain Atlas	Allen Brain Atlas	Bakker et al., 2015
**Other**		
Silicon probe; A2x2tet-10mm-150-150-121	NeuroNexus	http://neuronexus.com/
Jetball Dome	PhenoSys	https://www.phenosys.com/products/virtual-reality/jetball-dome/

CONTACT FOR REAGENT AND RESOURCE SHARING

Further information and requests for reagents and resource may be directed to the Lead Contact, Peter Somogyi (peter.somogyi@pharm.ox.ac.uk).

EXPERIMENTAL MODEL AND SUBJECT DETAILS

Extracellular electrophysiological recordings were performed in adult male C57Bl6/J mice using either multichannel silicon probes (n = 7 mice) or glass electrodes (n = 24 mice). At the time of surgery, mice ranged between 3 and 6 months of age. Mice were housed with littermates until surgical implantation of the head-plate, after which they were housed individually and maintained on a 12 hr light/dark schedule with lights off at 7:00 pm. All behavioral training and recording occurred in the light phase. All procedures involving experimental animals were under approved personal and project licenses in accordance with the Animals (Scientific Procedures) Act, 1986 (UK) and associated regulations.

METHOD DETAILS

Surgery

For surgical implantation, mice were deeply anesthetized with isoflurane (induction chamber 3% - 4% v/v with airflow and reduced to 1% - 2% v/v after the animal was positioned in the stereotaxic apparatus) and given a subcutaneous injection of buprenorphine (Vetergesic, 0.08 mg/kg). The skull was exposed under aseptic conditions, and three sites were marked: the bregma, MS craniotomy (antero-posterior (AP): +0.86 mm, medio-lateral (ML): 0 mm, dorso-ventral (DV): 3.5 mm, 0°) and hippocampal craniotomy (AP: −2.4 mm, ML: 1.7 mm, DV: 1.2 mm, 10° postero-anterior angle). The head-plate (either a 0.7g or 1.1g version, custom made at the Department of Physics, Oxford University) used for head-fixation was secured to the skull using dental cement and three small M1 screws. Two such screws were fixed to the skull above the cerebellum and served as the ground and electrical reference for the recordings.

Behavioral procedures

Upon recovery from the surgery (typically 3 to 4 days), mice were trained to run on an air-flow suspended styrofoam ball (for multi-channel silicon probes) or spherical treadmill (for single cell recordings). After recovery, food restriction (to 90% of initial pre-surgery body weight) was aimed to motivate running and mice received small drops of sucrose (20% solution) reward upon reaching the end of the linear maze on the styrofoam ball (jetball, PhenoSys). Mice that performed anticipatory licking and whose tail was balanced, as if running on a linear track, were considered trained. This training was a crucial behavioral control for each animal.

Recordings and single unit identification

The recording sessions lasted between 30 to 60 min in case of multi-channel silicon probe recordings and up to 3 hr for single cell recordings while aiming for neurons with a particular firing pattern. The activity of MS cells was recorded during multiple running (RUN) and resting (REST) periods. LFP theta oscillation was recorded in the pyramidal cell layer or in stratum oriens in CA1 and referenced to a screw in contact with the dura above the cerebellum, with 0° set as the trough. We defined this location in CA1 by positive going “sharp waves” in the LFP during REST, which were recognized by the co-occurrence of 130-230 Hz filtered “ripple” oscillations. Sharp waves appear as a negative potential in stratum radiatum. Wide-band (0.1 – 6000 Hz; 20 kHz sampling rate) recordings were performed using a 2 shank acute silicon probe (150 μm intershank distance; 2 tetrodes per shank; 25 μm spacing between contacts within a tetrode, Neuronexus) connected to a RA16-AC preamplifier (Tucker-Davies). Recordings were then digitally high pass filtered (0.8 – 5 kHz) and neuronal spikes were detected using a threshold crossing based algorithm. Detected spikes were automatically sorted using the algorithm implemented in KlustaKwik (Kadir et al., 2014), followed by manual adjustment of the clusters (Csicsvari et al., 1999) to obtain well-isolated single units, based on cross-correlations, spike-waveform and refractory periods. Multiunit or noise clusters or those with less than 300 spikes were discarded from analysis.

Juxtacellular labeling

Extracellular single-cell recording and juxtacellular labeling with neurobiotin reveals the identity of the labeled cells in conjunction with their *in vivo* activity patterns, target area, and synaptic targets. Using multi-unit recordings in awake head-fixed mice, we were able to identify the stereotyped activity patterns of Teevra neurons. Spikes of putative Teevra neurons were recorded during RUN and REST periods (Movie S1) using an extracellular glass electrode filled with 3% neurobiotin in 0.5 M NaCl and the spike output was converted into audio signal. Subsequently, if the neuron was deemed to be a Teevra cell, based on the “sound of the burst” corresponding to a short burst duration, and if the firing rate from REST to RUN did not appear to change, an attempt was made to label the cells with neurobiotin using the juxtacellular method (Pinault, 1996). We found four classes of MS rhythmic neurons. In addition to the most numerous Teevra cells, the second group, which we named *Komal* (“soft sound of burst”) could reliably be differentiated from Teevra cell based on their long burst duration and increase in firing rate during RUN. The potential success of juxtacellular labeling is predicted by analyzing cellular health post-labeling *in vivo*, by comparing the spiking of the cells before and after the firing modulation

attempt. Successful labeling attempts were defined as those in which the cell was modulated for more than 30 s and action potentials could still be seen and heard after the modulation attempt. In such cases, neurobiotin was left to be transported in the neurons for 4 to 8 hr to allow for potential terminal bouton labeling, which was assessed after processing the brain.

Tissue processing and immunohistochemistry

Mice were deeply anesthetized with sodium pentobarbital (50 mg/kg, i.p.) and transcardially perfused with saline followed by a fixative solution (4% paraformaldehyde, 15% v/v saturated picric acid, 0.05% glutaraldehyde in 0.1 M PB at pH 7.4). Some brains were postfixed overnight in glutaraldehyde-free fixative. After washing in 0.1 M PB, coronal sections (70-80 μm) were cut using a vibratome (Leica VT 1000S, Leica Microsystems) and stored in 0.1M PB with 0.05% sodium azide at 4°C for further processing. The neurobiotin-labeled processes could be visualized in selected sets of sections using streptavidin-conjugated fluorophores in tissue sections previously permeabilized by Tris-buffered saline (TBS) with 0.3% Triton X-100 (TBS-Tx) or through rapid 2x freeze-thaw (FT) over liquid nitrogen (cryoprotected in 20% sucrose in 0.1 M PB). To visualize proteins within selected labeled cellular domains or in their synaptic targets, the sections were first blocked for 1 hr at room temperature (RT) in 20% normal horse serum (NHS) and then incubated in primary antibody solution containing 1% NHS for 2 to 4 days at 4°C. For the specificity of the method, negative controls which lacked the primary antibodies were processed in parallel. Primary antibodies (Key resource table) were detected with fluorophore-conjugated secondary antibodies for wide-field epifluorescence and confocal microscopy (Ferraguti et al., 2004; Lasztóczi et al., 2011). After primary antibody incubation, sections were washed three times for 10 min and transferred to a secondary antibody solution containing 1% NHS for 4 hr at RT or overnight at 4°C. Following secondary antibody incubation, sections were washed three times for 10 min each and mounted on glass slides in VectaShield. Our strategy exploits the distinct subcellular locations of target proteins (e.g nucleus, plasma membrane). Using this approach, the labeled process or synaptic target neurons could be simultaneously processed with 4 primary antibodies and subsequently multiple times if the cellular domain to be tested lacked detectable immunoreactivity in the previous round of reactions or if the molecule was not in the same cellular compartment. We were able to test the same cellular profile in a given section for up to 6 different molecules and different sections for up to 8 molecules of a single cell.

For light microscopic visualization, the neurobiotin signal was amplified by incubating the TBS-Tx or FT processed sections in avidin-biotin complex (VECTASTAIN Elite ABC HRP Kit) for 3 to 7 days depending on the strength of labeling (incubating for longer duration sometimes improved the neurobiotin visualization). The sections were then processed using horseradish peroxidase based diaminobenzidine (DAB) reactions, using the glucose oxidase method for the generation of H_2O_2 and nickel-intensified DAB as chromogen. The sections were osmium tetroxide treated (0.5%–1% in 0.1 M PB), sequentially dehydrated, and mounted on slides in epoxy resin (Unal et al., 2015). Selected sections for electron microscopy were incubated in 2% wt/vol uranyl acetate during dehydration.

Anatomical data analysis

Light and florescence microscopy

Wide field epifluorescence microscopy and confocal microscopy (Carl Zeiss LSM 710) were used to evaluate antibody reacted sections. Sections were first observed with a wide field epifluorescence microscope (Leitz DMRB; Leica Microsystems) equipped with PL Fluotar objectives. Multichannel fluorescence images were acquired with ZEN 2008 software v5.0 on a Zeiss LSM 710 laser scanning confocal microscope (Zeiss Microscopy), equipped with DIC M27 Plan-Apochromat 40/1.3 numerical aperture, DIC M27 Plan Apochromat 63/1.4 numerical aperture, and Plan-Apochromat 100/1.46 numerical aperture oil-immersion objectives. The following channel specifications were used (laser/excitation wavelength, beam splitter, emission spectral filter) for detection of Alexa405, Alexa488/EYFP, Cy3, and Cy5: 405-30 solid-state 405 nm with attenuation filter ND04, MBS-405, 409–499 nm; argon 488 nm, MBS-488, 493–542 nm; HeNe 543 nm, MBS-458/543, 552–639 nm; HeNe 633 nm, MBS-488/543/633, 637–757 nm. The pinhole size was set to 1 Airy unit for the shortest wavelength while, to keep all channels at the same optical slice thickness, the pinhole sizes of the longer wavelength channels were adjusted to values close to 1 Airy unit. Thus, optical section thickness for all channels was based on the set pinhole size for the shortest wavelength channel.

The osmium-treated sections mounted in resin after the HRP enzyme reaction (DAB) were analyzed using transmitted light microscope. To reveal the axonal arbor of Teevra cells in the CA3 and sample boutons in different regions and layers, two-dimensional reconstructions were made with a drawing tube attached to the transmitted light microscope equipped with a 63X oil-immersion objective. For all visualized axons, the first and last antero-poterior section containing boutons and the last section with the axon was established.

Electron microscopy

We tested the correspondence of light microscopically predicted putative synapses between target cells and axonal varicosities by electron microscopy of selected axon collaterals in osmium-treated sections. This enabled us to determine the probability of determining synaptic junctions based on axonal swellings next to a target profile. Serial sections (70 nm) were cut and mounted on single-slot, pioloform-coated copper grids for conventional transmission electron microscopy. Images were acquired with a Gatan UltraScan 1000 CCD camera. All neurobiotin containing boutons cut at the section plane were followed in serial sections to locate synaptic junctions. We identified synapses as Gray's type I (often called asymmetrical) and type II (often called symmetrical) based on their fine structure; type I synapses having a thick postsynaptic density, whereas type II synapses are characterized by a thin postsynaptic density (Gray, 1959).

Calculation of 3D distance in the hippocampus

We used two estimates to compute the distance along the hippocampus where the first branch or branch with boutons was observed, (1) the linear distance of the soma section to the section containing the first axonal collateral in the hippocampus as a measure of the anterior-posterior distance from the septum and, (2) using the Scalable Brain Atlas (Bakker et al., 2015) we approximated the medio-lateral, antero-posterior and dorso-ventral coordinates of the spatial location in the hippocampus to compute the 3D Euclidian distance of each coordinate from the septal pole of the hippocampal formation (ML: 0; AP: −0.98; DV: −1.4). These distances were highly correlated (linear correlation coefficient: $r = 0.89$, $p = 0.003$).

Electrophysiological data analysis

Data were analyzed in MATLAB (R2014a, MathWorks) and Spike2 (CED). RUN was defined as locomotion, whereby the jetball (in virtual reality) or circular treadmill (for single cell experiments, Table 1) was physically advanced by the mouse, based on virtual reality feedback or video observations. For single cell experiments, this additionally included signals from a motion sensor within the circular treadmill. The initiation of locomotion was also included in RUN. Small postural shifts in the absence of limb motion were excluded from RUN. For labeled Teevra cells MS71c, MS73c, and MS90g, in addition to video analysis, signals from an external accelerometer in contact with a running disc were used to define RUN. REST periods included everything outside RUN periods. There was no or little contamination from sleep as the animals were mostly awake and alert even during REST periods. To detect the zero crossings of the LIA, the CA1 reference LFP channel was downsampled to 1 kHz, the signal was rectified (time constant: 0.08 s) and the zero crossings were detected at falling level (minimum interval: 200 ms).

Rhythmicity Index

Rhythmicity index is based on the "theta index" (Royer et al., 2010), but it has fewer parameters (3 versus 6 in Royer et al.), for a more robust fit. Data were prepared by calculating the spike time autocorrelogram (bin width 10 ms, maximum lag 500 ms) for spikes defined in periods of RUN or REST. Next, the autocorrelogram was normalized by dividing the peak value between 100 and 200 ms (range chosen to match theta frequency first side band), and center values were clipped so that overall maximum is 1. We then fit a linear trend line to the above (dotted line in the figures) and perform a nonlinear fit (using MATLAB lsqnonlin function) to the detrended data. The fitting function is a Gaussian-modulated cosine function with three parameters: (1) cosine (theta) frequency in Hz (between 4 and 8); (2) the peak value of the Gaussian scaling function (high value indicates strong short-latency theta modulation) and (3) standard deviation (width) of Gaussian scaling function (high value indicates prolonged theta modulation). The solid red lines in Figures 2 and 6 are the fitted sinusoid functions (oscillatory frequency of the neuron), and the trends are shown by dotted lines. A coefficient of determination is measured at this stage to measure the goodness of fit. After fitting, the rhythmicity index is calculated as follows: (1) for each peak and trough in 50 to 500 ms, the absolute value of the fitted sinusoid is divided by the corresponding trend line value (between zero and one) and (2) the rhythmicity index is taken as the mean of these trend-normalized peak values.

Burst duration

Spikes were gathered by growing group such that nearest neighbor spikes were iteratively added unless interspike interval (ISI) > threshold; when both neighboring ISIs are > threshold burst was delimited; min spikes = 2, threshold = mean ISI. Mean burst durations during RUN are reported throughout.

Rate change score and speed correlation

Mean firing rates (Hz) were calculated during RUN and REST periods. A rate change score was then computed according to the following formula:

$$\text{rate change score} = \frac{\text{rate(RUN)} - \text{rate(REST)}}{\text{rate(RUN)} + \text{rate(REST)}}$$

Score ranges from −1 (decrease of rate during running) to 1 (increase of rate during running). The speed modulation of MS unit firing was also calculated for each neuron recorded using multi-channel probes. Data were binned at 1cm/s. For each speed bin (> 2cm/s), the corresponding firing frequency was calculated as the number of detected action potentials while the mouse was moving at this speed, divided by the duration the mouse ran at this speed. The firing frequencies per speed bin were linearly fitted with a weighting function for each bin equal to the square root of its duration. A linear correlation coefficient was computed for each neuron and it was determined if the firing is positively modulated ($r > 0$, $p < 0.05$), negatively modulated ($r < 0$, $p < 0.05$) or not correlated with the running speed of the animal ($p > 0.05$).

Mean firing phase preference

For each recorded neuron, we determined the mean depth of theta modulation and the preferential mean theta phase of firing (circular mean ± circular SD) using Rayleigh's method (Zar, 1999).

Hierarchical clustering

All analyses were performed using "Cluster Analysis" toolbox in MATLAB (R2014a, MathWorks). All parameters (m) for all neurons (n) are prepared in an "m-by-n" data matrix. The data are linearly rescaled and set to a new defined min and max (−1 to 1, rescale function, MATLAB) and stored in data matrix (e.g., X). Next, we calculate the pairwise Euclidian distance between each pair of observations in X (using MATLAB "pdist" function) and store this in a matrix (e.g., D). Next, we calculate the linkages and create a hierarchical

cluster tree. MATLAB function linkage returns a tree (e.g., Z) that encodes hierarchical clusters of the real matrix X using "Ward" method to measure the distance between clusters. A tree encoded by Z is plotted (using MATLAB function "dendrogram") and four major branches are selected (using MATLAB function "cluster") and the smallest height at which a horizontal cut through the tree leaves 4 clusters is identified (using MATLAB function "maxclust"). The cluster value assignment for each neuron is stored in a matrix (e.g., C). The silhouette method is used to determine the goodness of clustering. The silhouette value (MATLAB, Cluster Analysis toolbox, range: -1 to 1) for each point is a measure of how similar that point is to points in its own cluster versus points in other clusters according to the following formula:

$$Si = (bi - ai)/\max(ai, bi)$$

where a_i is the average distance from the ith point to the other points in the same cluster as i, and b_i is the minimum average distance from the ith point to points in a different cluster, minimized over clusters. Silhouette value for a cluster is reported as the mean silhouette of its individual members. Large positive values indicate that the cluster is compact and distinct from other clusters.

QUANTIFICATION AND STATISTICAL ANALYSIS

Standard functions and custom-made scripts in MATLAB were used to perform all analysis. We have not estimated the minimal population sample for statistical power, but the number of animals and labeled neurons were similar to or larger than those employed in previous works (Katona et al., 2014; Varga et al., 2014; Viney et al., 2013). The data were tested for normal distribution. Parametric tests were used for normally distributed data and non-parametric tests were applied to all other data. For a comparisons of firing phase preferences of different cell types, we used Watsons U2 test. Kruskal-Wallis one-way analysis of variance was used to compare two groups. Box-plots represent median and 25th – 75th percentiles and their whiskers show data range. Outliers are shown as a "+"sign.

DATA AND SOFTWARE AVAILABILITY

The software used for data acquisition and analysis is available for download. Data will be made available upon request.

Neuron, Volume *96*

Supplemental Information

Behavior-Dependent Activity and Synaptic Organization of Septo-hippocampal GABAergic Neurons Selectively Targeting the Hippocampal CA3 Area

Abhilasha Joshi, Minas Salib, Tim James Viney, David Dupret, and Peter Somogyi

Supplemental Information

Cell ID	Target ID	Target Location	Domain	PV	SATB1	Endo-biotin	CCK	CB	SM	Figure
AJ42m	s47_d1	CA3	d	d+	u	u	u	u	u	
	s47_d2	CA3	d*	s, d +	n-	s+	s-	s-	s-	
	s47_d3	CA3	d*	s, d +	n+	s+	s-	s-	s-	
	s47_d4	CA3	d*	s, d +	n-	s+	s-	s-	s-	
	s47_d5	CA3	d	d+	u	u	u	u	u	
	s47_s1	CA3	s	s-	s-	s+	s+	s-	s-	5B
	s47_s2	CA3	s	s-	s-	s+	s+	s-	s-	5B
	s47_s3	CA3	s	s-	s-	s+	s+	s-	s-	
	s47_s4	CA3	s	s-	s-	s+	s+	s-	s-	
	s48_a1	CA3	s, d	s+	n-	s+	s-	u	u	S2 B
	s48_a4	CA3	s, d	s+	n-	s+	s-	u	u	5A
	s48_a8	CA3	s, d	s+	n-	s+	s-	u	u	5A
	s48_a9	CA3	s, d	s+	n-	s+	s-	u	u	5A
	s49_a1	CA3	s, d	s+	n-	s+	s-	u	u	S2 A
	s49_a2	CA3	s, d	s+	n-	s+	s-	u	u	
	s50_d1	CA3	d	d+	u	u	u	d-	u	
	s50_d2	CA3	d	d+	u	u	u	d-	u	
	s50_d3	CA3	d	d+	u	u	u	d-	u	
	s50_d4	CA3	d	d+	u	u	u	d-	u	
	s50_d5	CA3	d	d+	u	u	u	d-	u	
	s50_d7	CA3	d	d+	u	u	u	d-	u	
	s50_s1	CA3	s	s+	n-	s+	u	s-	u	S2 C
	s71_a10	MS	s	s+	u	s+	u	u	u	
	s71_a11	MS	s	s+	u	s+	u	u	u	
	s71_a9	MS	s, d	s, d +	n+	s+	u	u	u	3D
	s72_a1	MS	s, d	s, d +	n+	s+	u	u	u	
	s72_a2	MS	s, d	s, d +	n+	s+	u	u	u	
	s72_a3	MS	d	d+	u	u	u	u	u	
	s72_a4	MS	d	d+	u	u	u	u	u	
	s72_a5	MS	d	d-	u	u	u	u	u	
	s72_a6	MS	s	s+	n+	s+	u	u	u	
	s72_a7	MS	s	s+	n+	s+	u	u	u	
	s72_a8	MS	d	d+	u	u	u	u	u	
MS90g	s28_s1	CA3	s	s+	n-	s+	s-	u	u	
	s29_s1	CA3	s	s+	n-	s+	s-	u	u	
	s30_s1	CA3	s	s+	n-	s+	s-	u	u	
	s30_s2	CA3	s	s+	n-	s+	s-	u	u	
	s31_s2	CA3	sd	s+	n-	s+	s-	u	u	
	s32_s1	CA3	s	s+	n-	s+	s-	u	u	

Table S1. Subcortical and cortical synaptic target neurons of Teevra cells, AJ42m and MS90g. Molecular expression profiles of postsynaptic neurons, based on close apposition of septal terminals. '+' detectable positive immunoreactivity or signal; '-' undetectable immunoreactivity or signal in vicinity of immunopositive signals observed within subcellular domain: s, soma; n, nucleus; d, dendrite; d*, dendrite could be followed to the soma from which it originates. u, unknown (untested or unavailable).

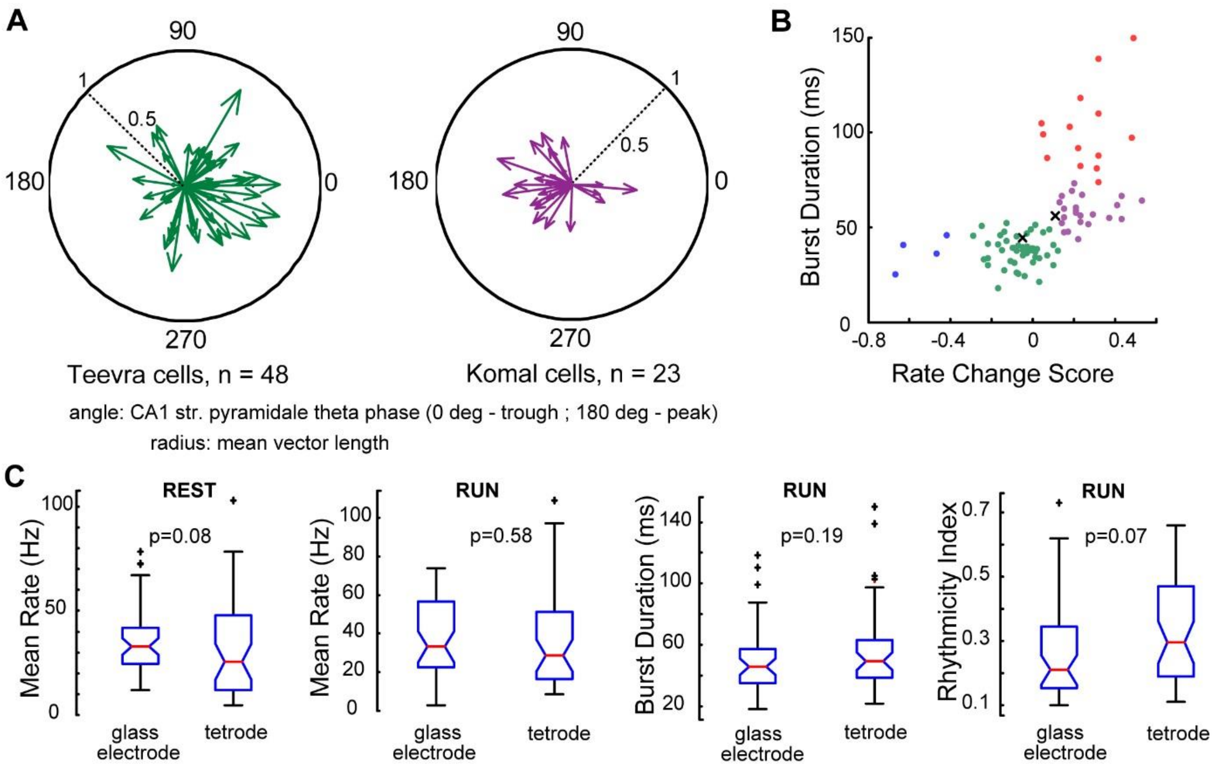

Figure S1: Physiological parameters of MS neurons recorded using glass-electrodes and silicon probes (related to Figure 1)

(A) Distribution of the mean theta firing phase of Teevra (green) and Komal (magenta) cells with mean vector length as radius. **(B)** Burst duration (ms) and rate change score of labelled Teevra neurons MS90g (x, left) and MS11b(x, right) in relation to other cells. These two neurons were not included in the clustering because the animals did not exhibit voluntary movement (see methods); the parameters were calculated during short bouts of theta oscillatory activity (Table 1). **(C)** Comparison of parameters calculated based on the spike train dynamics of neurons recorded using glass electrode or silicon probe recordings ($p > 0.05$, Kruskal-Wallis test). +, outliers.

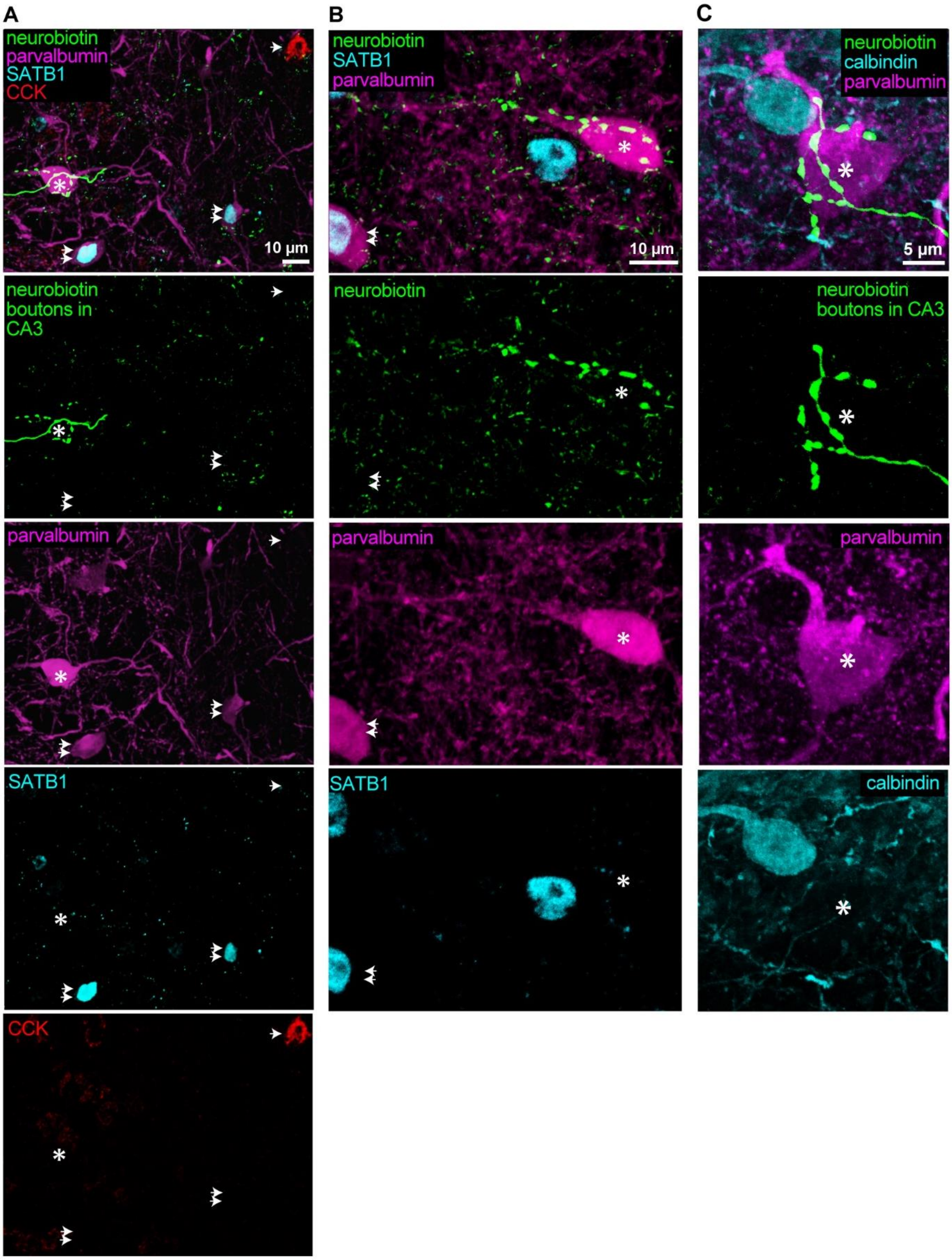

Figure S2: Postsynaptic PV+/SATB1-negative interneurons are consistently and preferentially innervated by Teevra cells. (related to Figure 5)

(A) Axonal terminals (green) of Teevra cell innervate PV+ (magenta, asterisks) and SATB1-negative (cyan) cells in a basket-like formation and also following their dendrites. Nearby PV+ and SATB1+ cells (double arrows) and a CCK+ cell (top, single arrow) were not innervated. **(B)** Axonal terminals (green) of Teevra cell innervate a PV^+ and SATB1-immunonegative cell in CA1 (labeling as in A). **(C)** Axonal terminals (green) of Teevra cell in CA3, innervate a PV+ (magenta, asterisk) and CB-negative (cyan) cell in a basket-like formation and also follow its dendrite, but do not innervate a neighboring CB^+ cell (cyan). The immunoreactivity of targeted interneurons is consistent with the molecular profile of axo-axonic cells. Imaging details (z-thickness in µm, z-projection type stated): (A) 9.74 µm, standard deviation projection (B) 11.7 µm, standard deviation projection (C) 14.70 µm, maximum intensity projection

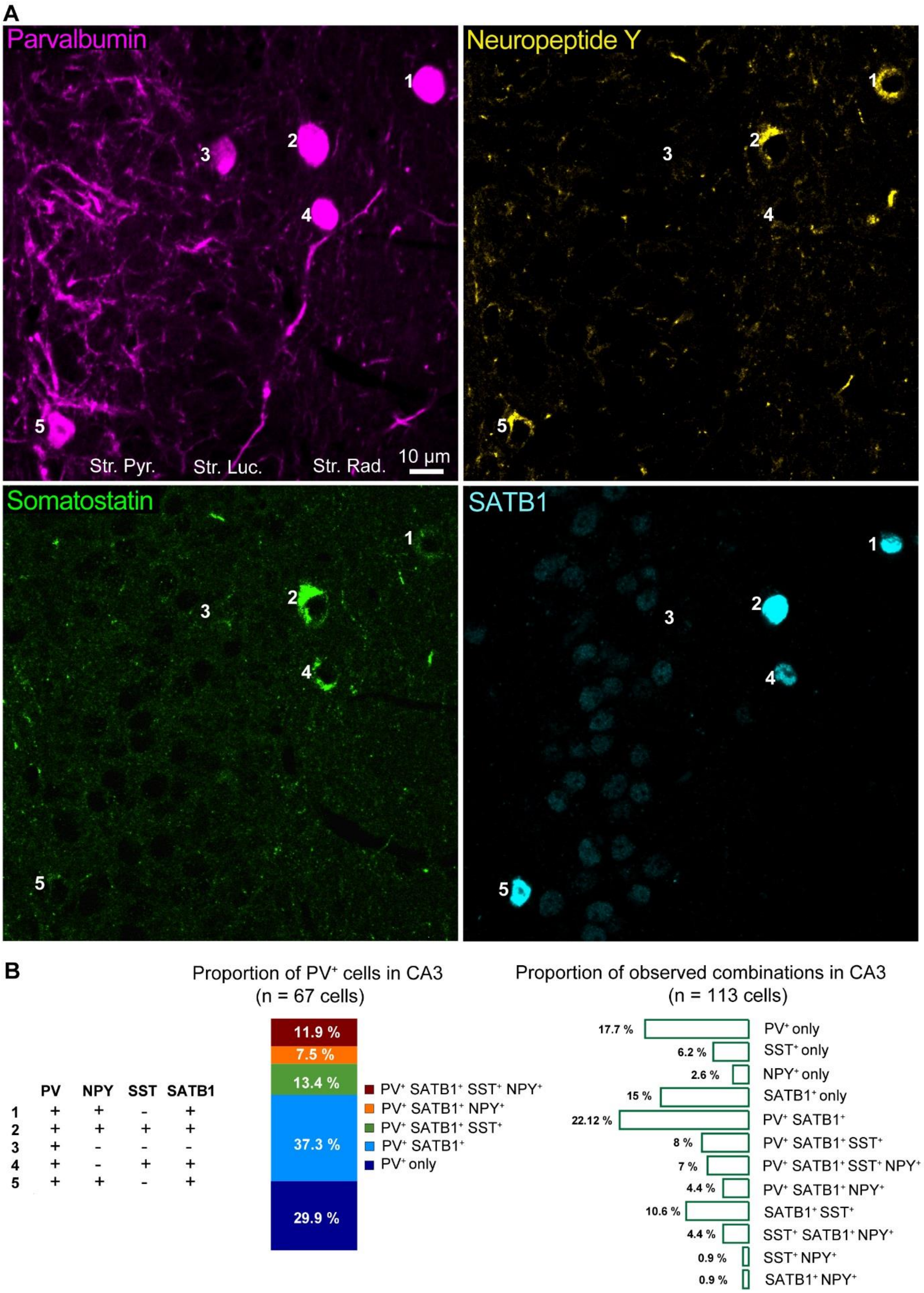

Figure S3: Molecular diversity of PV-expressing interneurons in mouse CA3 (related to Figure 5)

(A) A cluster of PV^+ interneurons (magenta, 1 to 5) exhibit a diverse combination of immunoreactivity for molecules expressed in strata pyramidale and lucidum of the mouse CA3. The SATB1-immunonegative nucleus of cell 3 is within the displayed optical slice and was also checked in other slices. **(B)** Left, immunoreactivity scores of the cells in 'A'. Middle, quantification of PV+ cells sampled with immunoreactivity for SATB1, NPY and SST (n = 3 mice, 5 areas). Note that PV-immunopositive and SATB1-immunonegative axo-axonic cells (Viney et al., 2013), comprise 30% of the PV+ population. All other PV+ cells also are SATB1+ in combination with other molecular markers such as SST and NPY. Right, proportions of interneurons labeled for combinations of the 4 molecules. Imaging details (z-thickness): 0.38 µm, single optical section.
